# Supplementary material for: Mapping retracted articles and exploring regional differences in China, 2012–2023
Source: PLoS One. 2024 Dec 2;19(12):e0314622. doi: 10.1371/journal.pone.0314622 (PMC11611127; doi:10.1371/journal.pone.0314622)
Supplement: S1 Table — (DOCX) [file pone.0314622.s001.docx]

**S1 Table.** **The number of publications (Retraction rate) by Chinese authors from 2012-2023 based on Scopus and Web of Science**

| **Year** | **The number of publications (Retraction rate)** | |
| --- | --- | --- |
|  | **Web of Science** | **Scopus** |
| 2012 | 202832（0.12%） | 417,455（0.06%） |
| 2013 | 240900（0.16%） | 457,394（0.08%） |
| 2014 | 276009（0.05%） | 489,452（0.03%） |
| 2015 | 308850（0.11%） | 466,722（0.07%） |
| 2016 | 341298（0.10%） | 505,047（0.07%） |
| 2017 | 377792（0.10%） | 551,119（0.07%） |
| 2018 | 436305（0.07%） | 612,763（0.05%） |
| 2019 | 536132（0.09%） | 726,245（0.06%） |
| 2020 | 616085（0.15%） | 807,322（0.12%） |
| 2021 | 727457（0.29%） | 901,191（0.23%） |
| 2022 | 883953（0.35%） | 1,043,351（0.30%） |
| 2023 | 885338（0.64%） | 1,073,392（0.53%） |
