# Supplementary material for: Mapping retracted articles and exploring regional differences in China, 2012–2023
Source: PLoS One. 2024 Dec 2;19(12):e0314622. doi: 10.1371/journal.pone.0314622 (PMC11611127; doi:10.1371/journal.pone.0314622)
Supplement: S2 Table — (DOCX) [file pone.0314622.s002.docx]

**S2 Table. The distribution of subject of retracted articles from Chinese authors between 2012 and 2023(N/%)**

| **Subject** | | **2012** | **2013** | **2014** | **2015** | **2016** | **2017** | **2018** | **2019** | **2020** | **2021** | **2022** | **2023** | **2012-2023** |
| --- | --- | --- | --- | --- | --- | --- | --- | --- | --- | --- | --- | --- | --- | --- |
| Physical Sciences (PHY) | **total** | 204/43.13% | 210/34.88% | 51/14.57% | 108/10.96% | 129/11.91% | 139/9.69% | 187/21.62% | 187/14.56% | 219/6.87% | 327/5.54% | 350/4.12% | 856/5.03% | 2967/7.12% |
|  | Astronomy | 0/0.00% | 0/0.00% | 0/0.00% | 0/0.00% | 0/0.00% | 0/0.00% | 0/0.00% | 0/0.00% | 0/0.00% | 0/0.00% | 0/0.00% | 1/0.12% | 1/0.04% |
|  | Astrophysics | 0/0.00% | 0/0.00% | 0/0.00% | 0/0.00% | 1/0.78% | 1/0.72% | 2/1.07% | 2/1.07% | 0/0.00% | 1/0.31% | 2/0.57% | 1/0.12% | 10/0.37% |
|  | Chemistry | 46/22.55% | 14/6.67% | 14/27.45% | 20/18.52% | 31/24.03% | 26/18.71% | 27/14.44% | 43/22.99% | 34/15.53% | 39/11.93% | 27/7.71% | 34/3.97% | 355/13.16% |
|  | Cosmology | 0/0.00% | 0/0.00% | 0/0.00% | 0/0.00% | 0/0.00% | 0/0.00% | 0/0.00% | 0/0.00% | 1/0.46% | 0/0.00% | 1/0.29% | 0/0.00% | 2/0.07% |
|  | Crystallography/  Spectroscopy | 30/14.71% | 3/1.43% | 2/3.92% | 3/2.78% | 1/0.78% | 4/2.88% | 16/8.56% | 10/5.35% | 15/6.85% | 16/4.89% | 6/1.71% | 7/0.82% | 113/4.19% |
|  | Energy | 11/5.39% | 5/2.38% | 0/0.00% | 3/2.78% | 0/0.00% | 3/2.16% | 14/7.49% | 11/5.88% | 3/1.37% | 13/3.98% | 25/7.14% | 114/13.32% | 202/7.49% |
|  | Engineering -Chemical | 7/3.43% | 8/3.81% | 2/3.92% | 10/9.26% | 9/6.98% | 18/12.95% | 18/9.63% | 18/9.63% | 28/12.79% | 27/8.26% | 30/8.57% | 35/4.09% | 210/7.79% |
|  | Engineering -Electrical | 22/10.78% | 10/4.76% | 0/0.00% | 10/9.26% | 10/7.75% | 14/10.07% | 25/13.37% | 20/10.70% | 14/6.39% | 22/6.73% | 39/11.14% | 93/10.86% | 279/10.34% |
|  | Engineering - General | 44/21.57% | 107/50.95% | 0/0.00% | 3/2.78% | 3/2.33% | 5/3.60% | 2/1.07% | 1/0.53% | 1/0.46% | 37/11.31% | 25/7.14% | 83/9.70% | 311/11.53% |
|  | Engineering  Mechanical | 6/2.94% | 9/4.29% | 2/3.92% | 4/3.70% | 7/5.43% | 1/0.72% | 12/6.42% | 9/4.81% | 3/1.37% | 13/3.98% | 20/5.71% | 52/6.07% | 138/5.12% |
|  | Engineering - Structural | 3/1.47% | 4/1.90% | 2/3.92% | 3/2.78% | 3/2.33% | 8/5.76% | 6/3.21% | 5/2.67% | 10/4.57% | 14/4.28% | 21/6.00% | 50/5.84% | 129/4.78% |
|  | Geology | 1/0.49% | 5/2.38% | 3/5.88% | 8/7.41% | 5/3.88% | 4/2.88% | 2/1.07% | 5/2.67% | 13/5.94% | 16/4.89% | 9/2.57% | 22/2.57% | 93/3.45% |
|  | Hydrology | 4/1.96% | 4/1.90% | 0/0.00% | 2/1.85% | 1/0.78% | 0/0.00% | 2/1.07% | 1/0.53% | 1/0.46% | 1/0.31% | 2/0.57% | 1/0.12% | 19/0.70% |
|  | Materials Science | 12/5.88% | 18/8.57% | 11/21.57% | 19/17.59% | 27/20.93% | 31/22.30% | 20/10.70% | 20/10.70% | 27/12.33% | 34/10.40% | 45/12.86% | 160/18.69% | 424/15.72% |
|  | Mathematics | 6/2.94% | 8/3.81% | 7/13.73% | 13/12.04% | 9/6.98% | 7/5.04% | 8/4.28% | 10/5.35% | 24/10.96% | 33/10.09% | 27/7.71% | 70/8.18% | 222/8.23% |
|  | Nanotechnology | 3/1.47% | 3/1.43% | 1/1.96% | 2/1.85% | 9/6.98% | 3/2.16% | 5/2.67% | 16/8.56% | 24/11.42% | 25/7.65% | 44/12.57% | 94/10.98% | 230/8.53% |
|  | Physics | 8/3.92% | 9/4.29% | 6/11.76% | 6/5.56% | 13/10.08% | 14/10.07% | 23/12.30% | 13/6.95% | 25/7.31% | 33/10.09% | 17/4.86% | 13/1.52% | 171/6.34% |
|  | Statistics | 1/0.49% | 3/1.43% | 1/1.96% | 2/1.85% | 0/0.00% | 0/0.00% | 5/2.67% | 3/1.60% | 16/1.83% | 3/0.92% | 10/2.86% | 26/3.04% | 58/2.15% |
| Humanities (HUM) | **total** | 1/0.21% | 0/0.00% | 0/0.00% | 0/0.00% | 1/0.09% | 1/0.07% | 2/0.23% | 0/0.00% | 1/0.03% | 19/0.32% | 103/1.21% | 394/2.32% | 522/1.25% |
|  | Architecture | 1/100.00% | 0/0.00% | 0/0.00% | 0/0.00% | 0/0.00% | 0/0.00% | 0/0.00% | 0/0.00% | 0/0.00% | 3/15.79% | 6/5.83% | 24/6.09% | 35/6.70% |
|  | Arts - Film Studies | 0/0.00% | 0/0.00% | 0/0.00% | 0/0.00% | 0/0.00% | 0/0.00% | 0/0.00% | 0/0.00% | 0/0.00% | 0/0.00% | 3/2.91% | 10/2.54% | 13/2.49% |
|  | Arts - General | 0/0.00% | 0/0.00% | 0/0.00% | 0/0.00% | 0/0.00% | 0/0.00% | 0/0.00% | 0/0.00% | 0/0.00% | 7/36.84% | 34/33.01% | 194/49.24% | 235/45.02% |
|  | Arts - Music | 0/0.00% | 0/0.00% | 0/0.00% | 0/0.00% | 0/0.00% | 0/0.00% | 0/0.00% | 0/0.00% | 0/0.00% | 5/10.53% | 42/40.78% | 112/28.43% | 156/29.89% |
|  | Arts -Literature/Poetry | 0/0.00% | 0/0.00% | 0/0.00% | 0/0.00% | 0/0.00% | 0/0.00% | 0/0.00% | 0/0.00% | 0/0.00% | 1/5.26% | 10/9.71% | 25/6.35% | 36/6.90% |
|  | Cartography | 0/0.00% | 0/0.00% | 0/0.00% | 0/0.00% | 1/100.00% | 0/0.00% | 0/0.00% | 0/0.00% | 0/0.00% | 0/0.00% | 1/0.97% | 0/0.00% | 2/0.38% |
|  | History - Africa | 0/0.00% | 0/0.00% | 0/0.00% | 0/0.00% | 0/0.00% | 0/0.00% | 0/0.00% | 0/0.00% | 0/0.00% | 0/0.00% | 1/0.97% | 0/0.00% | 1/0.19% |
|  | History - Asia | 0/0.00% | 0/0.00% | 0/0.00% | 0/0.00% | 0/0.00% | 0/0.00% | 0/0.00% | 0/0.00% | 1/100.00% | 5/26.32% | 2/1.94% | 10/2.54% | 19/3.64% |
|  | History - Europe | 0/0.00% | 0/0.00% | 0/0.00% | 0/0.00% | 0/0.00% | 0/0.00% | 0/0.00% | 0/0.00% | 0/0.00% | 0/0.00% | 0/0.00% | 1//0.25% | 2/0.38% |
|  | History - General | 0/0.00% | 0/0.00% | 0/0.00% | 0/0.00% | 0/0.00% | 0/0.00% | 0/0.00% | 0/0.00% | 0/0.00% | 0/0.00% | 1/0.97% | 1/0.25% | 2/0.38% |
|  | Journalism | 0/0.00% | 0/0.00% | 0/0.00% | 0/0.00% | 0/0.00% | 0/0.00% | 0/0.00% | 0/0.00% | 0/0.00% | 1/5.26% | 1/0.97% | 12/3.05% | 14/2.68% |
|  | Philosophy | 0/0.00% | 0/0.00% | 0/0.00% | 0/0.00% | 0/0.00% | 0/0.00% | 0/0.00% | 0/0.00% | 0/0.00% | 0/0.00% | 1/0.97% | 4/1.02% | 5/0.96% |
|  | Religion | 0/0.00% | 0/0.00% | 0/0.00% | 0/0.00% | 0/0.00% | 0/0.00% | 0/0.00% | 0/0.00% | 0/0.00% | 0/0.00% | 1/0.97% | 1/0.25% | 2/0.38% |
| Environmental Sciences (ENV) | **total** | 13/2.75% | 12/1.99% | 11/3.14% | 14/1.42% | 14/1.29% | 15/1.05% | 25/2.89% | 15/1.17% | 14/0.44% | 281/4.76% | 74/0.87% | 268/1.58% | 756/1.81% |
|  | Climate Change | 0/0.00% | 0/0.00% | 0/0.00% | 0/0.00% | 1/7.14% | 0/0.00% | 0/0.00% | 0/0.00% | 0/0.00% | 8/2.85% | 1/1.35% | 4/1.49% | 14/1.85% |
|  | Climatology | 0/0.00% | 1/8.33% | 4/9.09% | 0/0.00% | 0/0.00% | 0/0.00% | 4/16.00% | 1/6.67% | 2/14.29% | 48/17.08% | 3/4.05% | 0/0.00% | 60/7.94% |
|  | Ecology | 1/7.69% | 2/16.67% | 2/18.18% | 0/0.00% | 7/28.57% | 4/26.67% | 0/12.00% | 0/0.00% | 2/14.29% | 50/17.79% | 21/28.38% | 47/17.54% | 136/17.99% |
|  | Environmental Sciences | 9/69.23% | 6/50.00% | 4/36.36% | 7/50.00% | 4/42.86% | 7/46.67% | 12/48.00% | 10/66.67% | 9/64.29% | 131/46.62% | 38/51.35% | 177/66.04% | 416/55.03% |
|  | Food Science | 2/15.38% | 1/8.33% | 1/9.09% | 1/7.14% | 4/7.14% | 2/13.33% | 1/4.00% | 1/6.67% | 0/00.00% | 5/1.78% | 4/5.41% | 16/5.97% | 35/4.63% |
|  | Ground/Surface Water | 1/7.69% | 2/16.67% | 3/27.27% | 6/42.86% | 2/14.29% | 2/13.33% | 520.00% | 3/20.00% | 1/7.14% | 39/13.88% | 7/9.46% | 24/8.96% | 95/12.57% |
| Health Sciences (HSC) | **total** | 92/19.45% | 85/14.12% | 106/30.29% | 330/33.50% | 340/31.39% | 598/41.67% | 147/16.99% | 391/30.45% | 956/29.97% | 610/10.34% | 896/10.56% | 4388/25.80% | 8939/21.46% |
|  | Alternative | 4/4.35% | 1/1.18% | 2/1.89% | 0/0.00% | 16/4.71% | 7/1.17% | 4/2.72% | 6/1.53% | 9/0.94% | 8/1.31% | 18/2.01% | 124/2.83% | 199/2.23% |
|  | Anesthesia/Anaesthesia | 2/2.17% | 1/1.18% | 1/0.94% | 3/0.91% | 4/1.18% | 2/0.33% | 2/1.36% | 1/0.26% | 9/0.94% | 8/1.31% | 14/1.56% | 71/1.62% | 118/1.32% |
|  | Biostatistics/Epidemiology | 0/00.00% | 1/1.18% | 1/0.94% | 8/2.42% | 1/0.29% | 8/1.34% | 6/4.08% | 8/2.05% | 13/1.36% | 8/1.31% | 13/1.45% | 46/1.05% | 113/1.26% |
|  | Cardiology | 5/5.43% | 9/10.59% | 7/6.60% | 13/3.94% | 21/6.18% | 7/1.17% | 5/3.40% | 10/2.56% | 9/0.94% | 7/1.15% | 6/0.67% | 30/0.68% | 129/1.44% |
|  | Cardiovascular | 3/3.26% | 4/4.71% | 12/11.32% | 17/5.15% | 24/7.06% | 12/2.01% | 5/3.40% | 10/2.56% | 30/3.14% | 14/2.30% | 55/6.14% | 366/8.34% | 552/6.18% |
|  | Dentistry | 3/3.26% | 1/1.18% | 1/0.94% | 3/0.91% | 3/0.88% | 2/0.33% | 1/0.68% | 1/0.26% | 3/0.31% | 2/0.33% | 9/1.00% | 28/0.64% | 57/0.64% |
|  | Dermatology | 1/1.09% | 1/1.18% | 2/1.89% | 2/0.61% | 3/0.88% | 2/0.33% | 2/1.36% | 0/0.00% | 11/1.15% | 5/0.82% | 3/0.33% | 31/0.71% | 63/0.70% |
|  | Diabetes | 0/0.00% | 1/1.18% | 1/0.94% | 10/3.03% | 4/1.18% | 4/0.67% | 2/1.36% | 3/0.77% | 7/0.73% | 12/1.97% | 13/1.45% | 69/1.57% | 126/1.41% |
|  | Drug Design | 3/3.26% | 4/4.71% | 0/0.00% | 9/2.73% | 14/4.12% | 39/6.52% | 0/0.00% | 3/0.77% | 5/0.52% | 16/2.62% | 28/3.13% | 34/0.77% | 155/1.73% |
|  | Endocrinology | 2/2.17% | 3/3.53% | 3/2.83% | 19/5.76% | 7/2.06% | 5/0.84% | 0/0.00% | 3/0.77% | 17/1.78% | 12/1.97% | 8/0.89% | 67/1.53% | 146/1.63% |
|  | Gastroenterology | 3/3.26% | 3/3.53% | 5/4.72% | 11/3.33% | 13/3.82% | 55/9.20% | 9/6.12% | 67/17.14% | 91/9.52% | 52/8.52% | 29/3.24% | 156/3.56% | 494/5.53% |
|  | General | 0/00.00% | 2/2.35% | 0/00.00% | 3/0.91% | 2/0.59% | 1/0.17% | 0/0.00% | 1/0.26% | 6/0.63% | 9/1.48% | 31/3.46% | 119 /2.71% | 174/1.95% |
|  | Geriatric | 0/00.00% | 0/00.00% | 1/0.94% | 2/0.61% | 1/0.29% | 1/0.17% | 0/0.00% | 1/0.26% | 2/0.21% | 4/0.66% | 7/0.78% | 68/1.55% | 87/0.97% |
|  | Immunology | 6/6.52% | 7/8.24% | 6/5.66% | 9/2.73% | 7/2.06% | 12/2.01% | 5/3.40% | 7/1.79% | 9/0.94% | 4/0.66% | 19/2.12% | 106/2.42% | 197/2.20% |
|  | Infectious Disease | 5/5.43% | 1/1.18% | 3/2.83% | 7/2.12% | 4/1.18% | 6/1.00% | 3/2.04% | 9/2.30% | 28/2.93% | 21/3.44% | 33/3.68% | 59/1.34% | 197/2.00% |
|  | Internal | 0/0.00% | 0/0.00% | 0/0.00% | 6/1.82% | 0/0.00% | 2/0.33% | 0/0.00% | 0/0.00% | 4/0.42% | 4/0.66% | 6/0.67% | 65/1.48% | 87/0.97% |
|  | Neurology | 12/13.04% | 8/9.41% | 10/9.43% | 17/5.15% | 17/5.00% | 30/5.02% | 3/2.04% | 7/1.79% | 40/4.18% | 24/3.93% | 47/5.25% | 204/4.65% | 419/4.69% |
|  | Nursing | 0/0.00% | 0/0.00% | 0/0.00% | 0/0.00% | 1/0.29% | 1/0.17% | 0/0.00% | 1/0.26% | 2/0.21% | 0/00.00% | 23/2.57% | 190/4.33% | 218/2.44% |
|  | Nutrition | 0/0.00% | 0/0.00% | 0/0.00% | 2/0.61% | 1/0.29% | 0/0.00% | 1/0.68% | 0/00.00% | 0/00.00% | 3/0.49% | 6/0.67% | 25/0.57% | 38/0.43% |
|  | Obstetrics/Gynecology | 2/2.17% | 0/0.00% | 6.60% | 11/3.33% | 7/2.06% | 17/2.84% | 5/3.40% | 16/4.09% | 70/7.32% | 33/5.41% | 35/3.91% | 207/4.72% | 410/4.59% |
|  | Occupational Health and Safety | 0/00.00% | 1/1.18% | 0/00.00% | 1/0.30% | 2/0.59% | 0/0.00% | 1/0.68% | 1/0.26% | 4/0.42% | 3/0.49% | 9/1.00% | 19/0.43% | 41/0.46% |
|  | Oncology | 8/8.70% | 5/5.88% | 14/13.21% | 54/16.36% | 60/17.65% | 143/23.91% | 10/6.80% | 75/19.18% | 312/32.64% | 140/22.95% | 108/12.05% | 466/10.62% | 1395/15.61% |
|  | Ophthalmology | 0/00.00% | 0/00.00% | 5/1.89% | 5/0.61% | 5/1.47% | 1/10.17% | 0/0.00% | 5/1.28% | 9/0.94% | 3/0.49% | 13/1.45% | 51/1.16% | 91/1.02% |
|  | Orthopedics | 2/2.17% | 0/00.00% | 2/1.89% | 21/6.36% | 6/1.76% | 20/3.34% | 10/6.80% | 17/4.35% | 50/5.23% | 35/5.74% | 35/3.91% | 183/4.17% | 381/4.26% |
|  | Otorhinolaryngology | 0/0.00% | 0/00.00% | 0/00.00% | 2/0.61% | 5/1.47% | 10/1.67% | 1/0.68% | 8/2.05% | 17/1.78% | 10/1.64% | 10/1.12% | 69/1.57% | 132/1.48% |
|  | Pathology | 0/0.00% | 2/2.35% | 2/1.89% | 14/4.24% | 27/7.94% | 101/16.89% | 3/2.04% | 0/00.00% | 3/0.31% | 0/0.00% | 3/0.33% | 1/0.02% | 156/1.75% |
|  | Pediatrics | 0/0.00% | 1/1.18% | 1/0.94% | 2/0.61% | 1/0.29% | 7/1.17% | /74.76% | 0/00.00% | 7/0.73% | 10/1.64% | 14/1.56% | 142/3.24% | 192/2.15% |
|  | Pharmacology | 3/3.26% | 9/10.59% | 8/7.55% | 12/3.64% | 28/8.24% | 19/3.18% | 9/6.12% | 8/2.05% | 19/1.99% | 23/3.77% | 49/5.47% | 236/5.38% | 423/4.73% |
|  | Psychiatry | 0/00.00% | 0/00.00% | 0/00.00% | 1/0.30% | 2/0.59% | 3/0.50% | 2/1.36% | 2/0.51% | 6/0.63% | 9/1.48% | 5/0.56% | 2/10.48% | 51/0.57% |
|  | Public Health and Safety | 3/3.26% | 3/3.53% | 1/0.94% | 5/1.52% | 4/1.18% | 4/0.67% | 6/4.08% | 5/1.28% | 10/1.05% | 25/4.10% | 28/3.13% | 86/1.96% | 180/2.01% |
|  | Pulmonology | 5/5.43% | 0/0.00% | 2/1.89% | 11/3.33% | 10/2.94% | 9/1.51% | 4/2.72% | 20/5.12% | 43/4/.50% | 34/5.57% | 32/3.57% | 176/4.01% | 346/3.87% |
|  | Radiology/Imaging | 7/7.61% | 5/5.88% | 1/0.94% | 6/1.82% | 5/1.47% | 10/1.67% | 6/4.08% | 8/2.05% | 28/2.93% | 15/2.46% | 47/5.25% | 231/5.26% | 369/4.13% |
|  | Rehabilitation/Therapy | 5/5.43% | 2/2.35% | 3/2.83% | 8/2.42% | 7/2.06% | 14/2.34% | 14/9.52% | 10/2.56% | 16/1.67% | 14/2.30% | 23/3.01% | 114/2.60% | 234/2.62% |
|  | Sports | 0/0.00% | 0/0.00% | 0/00.00% | 2/0.61% | 4/1.18% | 0/0.00% | 0/00.00% | 0/00.00% | 1/0.10% | 5/0.82% | 6/0.67% | 38/0.87% | 56/0.63% |
|  | Sports Science | 0/00.00% | 0/00.00% | 0/00.00% | 0/00.00% | 0/0.00% | 2/0.33% | 0/00.00% | 0/00.00% | 1/0.10% | 3/0.49% | 32/3.57% | 86/1.96% | 124/1.39% |
|  | Surgery | 3/3.26% | 5/5.88% | 8/7.55% | 19/5.76% | 16/4.71% | 18/3.01% | 12/8.16% | 40/10.23% | 19/1.99% | 14/2.30% | 48/5.36% | 278/6.34% | 480/5.37% |
|  | Transplantation | 0/00.00% | 0/00.00% | 0/00.00% | 0/00.00% | 0/00.00% | 2/0.33% | 0/00.00% | 28/7.16% | 1/0.10% | 1/0.16% | 2/0.22% | 6/0.14% | 40/0.45% |
|  | Urology/Nephrology | 3/3.26% | 5/2.35% | 0/00.00% | 16/4.85% | 5/1.47% | 22/3.68% | 7/4.76% | 10/2.56% | 43/4.50% | 19/3.11% | 22/2.46% | 118/2.69% | 267/2.99% |
|  | Veterinary Science | 2/2.17% | 3/3.53% | 0/00.00% | 2/0.61% | 3/0.88% | 0/0.00% | 2/1.36% | 0/0.00% | 2/0.21% | 1/0.16% | 3/0.33% | 2/0.05% | 20/0.22% |
| Basic Life Sciences (BLS) | **total** | 109/23.04% | 119/19.77% | 157/44.86% | 484/49.14% | 467/43.12% | 651/45.37% | 428/49.48% | 545/42.45% | 1857/58.21% | 4045/68.57% | 3767/43.32% | 4400/25.87% | 16938/40.66% |
|  | Agriculture | 3/2.75% | 1/0.84% | 0/0.00% | 1/0.21% | 7/1.50% | 2/0.31% | 2/0.47% | 5/0.92% | 3/0.16% | 36/0.89% | 38/1.03% | 77/1.75% | 175/1.03% |
|  | Anatomy/Physiology | 2/1.83% | 1/0.84% | 4/2.55% | 10/2.07% | 8/1.71% | 7/1.08% | 12/2.80% | 7/1.28% | 12/0.65% | 11/0.27% | 16/0.44% | 6/0.14% | 96/0.57% |
|  | Anthropology | 0/0.00% | 0/0.00% | 0/0.00% | 0/0.00% | 0/0.00% | 0/0.00% | 0/0.00% | 0/0.00% | 0/0.00% | 0/0.00% | 0/0.00% | 1/0.02% | 1/0.01% |
|  | Archeology | 0/0.00% | 0/0.00% | 0/0.00% | 0/0.00% | 0/0.00% | 0/0.00% | 0/0.00% | 0/0.00% | 0/0.00% | 0/0.00% | 1/0.03% | 1/0.02% | 2/0.01% |
|  | Biochemistry | 17/15.60% | 14/11.76% | 21/13.38% | 30/6.20% | 47/10.06% | 51/7.83% | 92/21.50% | 135/24.77% | 378/20.36% | 431/10.66% | 262/7.13% | 528/12.00% | 2006/11.84% |
|  | Cancer | 4/3.67% | 13/10.92% | 23/14.65% | 67/13.84% | 70/14.99% | 165/25.35% | 49/11.45% | 81/14.86% | 375/20.19% | 899/22.22% | 832/22.63% | 798/18.14% | 3376/19.93% |
|  | Cellular | 21/19.27% | 21/17.65% | 35/22.29% | 101/20.87% | 99/21.20% | 91/13.98% | 116/27.10% | 164/30.09% | 531/28.59% | 1133/28.01% | 1154/31.39% | 1172/26.64% | 4638/27.38% |
|  | Forensic Sciences | 0/0.00% | 0/0.00% | 0/0.00% | 0/0.00% | 0/0.00% | 1/0.15% | 0/0.00% | 0/0.00% | 0/0.00% | 1/0.02% | 2/0.05% | 0/0.00% | 4/0.02% |
|  | General | 4/3.67% | 8/6.72% | 3/3.82% | 15/3.10% | 11/2.36% | 7/1.08% | 1/0.23% | 0/0.00% | 3/0.16% | 7/0.17% | 53/1.44% | 37/0.84% | 152/0.90% |
|  | Genetics | 15/13.76% | 16/13.45% | 16/10.19% | 100/20.66% | 86/18.42% | 146/22.43% | 53/12.38% | 70/12.84% | 369/19.87% | 901/22.27% | 851/23.15% | 1012/23.00% | 3635/21.46% |
|  | Microbiology | 5/4.59% | 4/3.36% | 10/6.37% | 18/3.72% | 17/3.64% | 8/1.23% | 14/3.27% | 15/2.75% | 27/1.45% | 40/0.99% | 36/0.98% | 53/1.20% | 247/1.46% |
|  | Molecular | 18/16.51% | 22/18.49% | 26/16.56% | 108/22.31% | 77/16.49% | 120/18.43% | 44/10.28% | 5/0.92% | 33/1.78% | 327/8.08% | 155/4.22% | 352/8.00% | 1287/7.60% |
|  | Neuroscience | 5/5.50% | 4/5.04% | 10/5.10% | 18/2.69% | 17/5.14% | 8/2.92% | 14/1.17% | 13/2.39% | 31/1.67% | 47/1.16% | 38/1.03% | 56/1.27% | 266/1.57% |
|  | Nutrition | 0/0.00% | 2/1.68% | 0/0.00% | 3/0.62% | 4/0.86% | 2/0.31% | 2/0.47% | 2/0.37% | 1/0.05% | 3/0.07% | 3/0.08% | 9/0.20% | 31/0.18% |
|  | Paleontology | 0/0.00% | 0/0.00% | 0/0.00% | 1/0.21% | 0/0.00% | 0/0.00% | 0/0.00% | 0/0.00% | 0/0.00% | 1/0.02% | 0/0.00% | 1/0.02% | 3/0.02% |
|  | Parasitology | 1/0.92% | 0/0.00% | 0/0.00% | 0/0.00% | 0/0.00% | 1/0.15% | 1/0.23% | 1/0.18% | 2/0.11% | 0/0.00% | 4/0.11% | 2/0.05% | 12/0.07% |
|  | Plant Biology/Botany | 9/8.26% | 4/0.84% | 4/2.55% | 4/0.83% | 12/2.57% | 10/1.54% | 16/3.74% | 17/3.12% | 14/0.75% | 35/0.87% | 36/0.98% | 78/1.77% | 236/1.39% |
|  | Toxicology | 2/1.83% | 4/3.36% | 4/0.64% | 10/2.07% | 0/0.00% | 15/2.30% | 21/4.91% | 25/4.59% | 74/3.98% | 168/4.15% | 171/4.65% | 199/4.52% | 690/4.07% |
|  | Zoology | 2/1.83% | 6/5.04% | 3/1.91% | 3/0.62% | 5/1.07% | 6/0.92% | 0/0.00% | 5/0.92% | 4/0.22% | 5/0.12% | 24/0.65% | 18/0.41% | 81/0.48% |
| Business and Technology (B/T) | **total** | 48/10.15% | 136/22.59% | 19/5.43% | 39/3.96% | 112/11.27% | 23/1.60% | 59/6.82% | 126/9.81% | 116/3.64% | 495/8.39% | 2651/31.24% | 4631/27.23% | 8465/20.32% |
|  | Business - Accounting | 0/0.00% | 2/1.47% | 0/0.00% | 0/0.00% | 0/0.00% | 1/4.35% | 1/1.69% | 1/0.79% | 0/0.00% | 10/2.02% | 50/1.88% | 33/0.71% | 98/1.16% |
|  | Business - Economics | 3/6.25% | 7/5.15% | 3/15.79% | 3/7.69% | 3/2.46% | 1/4.35% | 3/5.08% | 3/2.38% | 2/1.72% | 38/7.68% | 127/4.79% | 271/5.85% | 464/5.48% |
|  | Business - General | 9/18.75% | 26/19.12% | 0/0.00% | 4/10.26% | 0/0.00% | 0/0.00% | 1/1.69% | 1/0.79% | 1/0.86% | 26/5.25% | 72/2.71% | 196/4.23% | 336/3.97% |
|  | Business - Management | 8/16.67% | 45/33.09% | 1/5.26% | 2/5.13% | 0/0.00% | 0/0.00% | 0/0.00% | 2/1.59% | 1/0.86% | 15/3.03% | 107/4.03% | 177/3.82% | 358/4.23% |
|  | Business - Manufacturing | 1/2.08% | 10/7.35% | 0/0.00% | 1/2.56% | 1/0.82% | 0/0.00% | 12/20.34% | 4/3.17% | 2/1.72% | 6/1.21% | 43/1.62% | 66/1.43% | 146/1.72% |
|  | Business – Marketing | 0/0.00% | 1/0.74% | 0/0.00% | 1/2.56% | 0/0.00% | 0/0.00% | 0/0.00% | 0/0.00% | 1/0.86% | 5/1.01% | 32/1.21% | 51/1.10% | 91/1.08% |
|  | Business –  Public Relations | 0/0.00% | 0/0.00% | 0/0.00% | /0.00% | 0/0.00% | 0/0.00% | 0/0.00% | 0/0.00% | 0/0.00% | 0/0.00% | 3/0.11% | 4/0.09% | 7/0.08% |
|  | Computer Science | 9/18.75% | 8/5.88% | 10/52.63% | 14/35.90% | 53/43.44% | 11/47.83% | 18/30.51% | 54/42.86% | 59/50.86% | 150/30.30% | 862/32.48% | 828/17.88% | 2076/24.52% |
|  | Data Science | 0/0.00% | 0/0.00% | 0/0.00% | 0/0.00% | 0/0.00% | 0/0.00% | 0/0.00% | 0/0.00% | 0/0.00% | 0/0.00% | 148/5.58% | 1367/29.52% | 1515/17.90% |
|  | Foreign Aid | 0/0.00% | 0/0.00% | 0/0.00% | 0/0.00% | 0/0.00% | 1/4.35% | 0/0.00% | 0/0.00% | 2/1.72% | 0/0.00% | 0/0.00% | 1/0.02% | 4/0.05% |
|  | Government | 0/0.00% | 0/0.00% | 1/5.26% | 2/5.13% | 0/0.00% | 1/4.35% | 1/1.69% | 0/0.00% | 1/0.86% | 4/0.81% | 16/0.60% | 36/0.78% | 62/0.73% |
|  | International Relations | 0/0.00% | 1/0.74% | 0/0.00% | 2/5.13% | 0/0.00% | 1/4.35% | 1/1.69% | 2/1.59% | 2/1.72% | 5/1.01% | 20/0.75% | 33/0.71% | 67/0.79% |
|  | Technology | 15/31.25% | /2820.59% | 4/21.05% | 10/25.64% | 63/51.64% | 5/21.74% | 17/28.81% | 51/40.48% | 36/31.03% | 187/37.78% | 1080/40.69% | 1375/29.69% | 2871/33.92% |
|  | Transportation | 0/0.00% | 4/2.94% | 0/0.00% | 0/0.00% | 1/0.82% | 1/4.35% | 2/3.39% | 4/3.17% | 4/3.45% | 13/2.63% | 36/1.36% | 86/1.86% | 151/1.78% |
|  | Urban Planning | 3/6.25% | 4/2.94% | 0/0.00% | 0/0.00% | 1/0.82% | 1/4.35% | 3/5.08% | 4/3.17% | 5/0.31% | 36/7.27% | 55/2.07% | 107/2.31% | 219/2.59% |
| Social Sciences (SOC) | **total** | 6/1.27% | 40/6.64% | 6/1.71% | 10/1.02% | 10/0.92% | 8/0.56% | 17/1.97% | 20/1.56% | 27/0.85% | 122/2.07% | 735/8.66% | 2069/12.17% | 3070/7.37% |
|  | Communications | 2/33.33% | 2/5.00% | 2/33.33% | 3/30.00% | 3/30.00% | 2/25.00% | 2/11.76% | 1/5.00% | 4/14.81% | 4/3.28% | 37/5.03% | 108/5.22% | 170/ 5.54% |
|  | Criminology | 0/0.00% | 0/0.00% | 0/0.00% | 0/0.00% | 0/0.00% | 0/0.00% | 0/0.00% | 0/0.00% | 0/0.00% | 0/0.00% | 2/0.27% | 7/0.34% | 9/0.29% |
|  | Education | 1/16.67% | 2/5.00% | 2/33.33% | 0/0.00% | 3/30.00% | 0/0.00% | 6/35.29% | 7/35.00% | 4/14.81% | 42/34.43% | 433/58.91% | 865/41.81% | 1365/44.46% |
|  | Ethics/Bioethics | 0/0.00% | 0/0.00% | 0/0.00% | 0/0.00% | 0/0.00% | 0/0.00% | 1/5.88% | 1/5.00% | 2/7.41% | 0/0.00% | 865/0.41% | 1/0.05% | 8/0.26% |
|  | Forensics | 0/0.00% | 0/0.00% | 0/0.00% | 0/0.00% | 0/0.00% | 0/0.00% | 0/0.00% | 1/5.00% | 2/7.41% | 1/0.82% | 1/0.14% | 1/0.05% | 6/0.20% |
|  | Law/Legal Issues | 0/0.00% | 0/0.00% | 0/0.00% | 0/0.00% | 0/0.00% | 0/0.00% | 0/0.00% | 1/5.00% | 0/0.00% | 2/1.64% | 14/1.90% | 37/1.79% | 54/1.76% |
|  | Linguistics | 0/0.00% | 1/2.50% | 0/0.00% | 0/0.00% | 0/0.00% | 0/0.00% | 0/0.00% | 0/0.00% | 0/0.00% | 6/4.92% | 35/4.76% | 196/9.47% | 238/7.75% |
|  | Military/Naval Studies | 0/0.00% | 29/72.50% | 0/0.00% | 0/0.00% | 0/0.00% | 0/0.00% | 0/0.00% | 0/0.00% | 0/0.00% | 1/0.82% | 1/0.14% | 2/0.10% | 33/1.07% |
|  | Philosophy | 0/0.00% | 0/0.00% | 0/0.00% | 0/0.00% | 0/0.00% | 0/0.00% | 0/0.00% | 0/0.00% | 0/0.00% | 4/3.28% | 1/0.14% | 12/0.58% | 17/0.55% |
|  | Political Science | 0/0.00% | 1/2.50% | 0/0.00% | 2/20.00% | 0/0.00% | 0/0.00% | 1/5.88% | 0/0.00% | 0/0.00% | 0/0.00% | 22/2.99% | 123/5.94% | 149/4.85% |
|  | Psychology | 3/50.00% | 3/7.50% | 1/16.67% | 2/20.00% | 1/10.00% | 5/62.50% | 4/23.53% | 5/25.00% | 9/33.33% | 21/17.21% | 43/5.85% | 265/12.81% | 362/11.79% |
|  | Sexual And Marital Studies | 0/0.00% | 0/0.00% | 0/0.00% | 0/0.00% | 0/0.00% | 0/0.00% | 1/5.88% | 0/0.00% | 4/3.70% | 2/1.64% | 1/0.14% | 0/0.00% | 5/0.16% |
|  | Sociology | 0/0.00% | 2/5.00% | 1/16.67% | 1/10.00% | 2/20.00% | 1/12.50% | 2/11.76% | 2/10.00% | 5/14.81% | 9/7.38% | 26/3.54% | 71/3.43% | 121/3.94% |
|  | Sports and Recreation | 0/0.00% | 0/0.00% | 0/0.00% | 2/20.00% | 1/10.00% | 0/0.00% | 0/0.00% | 2/10.00% | 1/3.70% | 30/24.59% | 116/15.78% | 381/18.41% | 533/17.36% |
