# Supplementary material for: Mapping retracted articles and exploring regional differences in China, 2012–2023
Source: PLoS One. 2024 Dec 2;19(12):e0314622. doi: 10.1371/journal.pone.0314622 (PMC11611127; doi:10.1371/journal.pone.0314622)
Supplement: S3 Table — (DOCX) [file pone.0314622.s003.docx]

**S3 Table. The top 10 publishers involved retracted articles from Chinese authors published between 2012 and 2023**

| **Publishers** | **Number of retracted articles** | **Proportion** |
| --- | --- | --- |
| Hindawi | 4464 | 30.90% |
| Springer | 2105 | 14.57% |
| Elsevier | 1407 | 9.74% |
| Taylor and Francis | 757 | 5.24% |
| Wiley | 691 | 4.78% |
| IOP Publishing | 577 | 3.99% |
| Spandidos | 457 | 3.16% |
| SAGE Publications | 444 | 3.07% |
| IEEE | 396 | 2.74% |
| Association for Computing Machinery (ACM) | 339 | 2.35% |
