# Supplementary material for: Mapping retracted articles and exploring regional differences in China, 2012–2023
Source: PLoS One. 2024 Dec 2;19(12):e0314622. doi: 10.1371/journal.pone.0314622 (PMC11611127; doi:10.1371/journal.pone.0314622)
Supplement: S4 Table — (DOCX) [file pone.0314622.s004.docx]

**S4 Table. The top 10 institutions involved retracted articles from Chinese first authors institutions between 2012 and 2023**

| **Institution*** | **Number of retracted articles** | **Proportion** |
| --- | --- | --- |
| China-Japan Union Hospital of Jilin University | 152 | 1.05% |
| The First Hospital of Jilin University | 90 | 0.62% |
| The First Affiliated Hospital of Zhengzhou University | 85 | 0.59% |
| Central South University | 64 | 0.44% |
| The Second Hospital of Jilin University | 50 | 0.35% |
| Jilin University | 49 | 0.34% |
| The Affiliated Hospital of Qingdao University | 48 | 0.33% |
| Zhejiang University | 46 | 0.32% |
| Sichuan University | 44 | 0.30% |
| University of Electronic Science and Technology of China | 44 | 0.30% |

Note: *The affiliation of the first author.
