# Supplementary material for: Mapping retracted articles and exploring regional differences in China, 2012–2023
Source: PLoS One. 2024 Dec 2;19(12):e0314622. doi: 10.1371/journal.pone.0314622 (PMC11611127; doi:10.1371/journal.pone.0314622)
Supplement: S5 Table — (DOCX) [file pone.0314622.s005.docx]

**S5 Table. Standardized reason categorization corresponding to categorization employed by the Retraction Watch Database**

| **Standardized reason categorization** | **Categorization employed by Retraction Watch Database** |
| --- | --- |
| Plagiarism | Plagiarism of Text, Plagiarism of Image, Plagiarism of Data, Plagiarism of Article, Euphemisms for Plagiarism |
| Fake Data | Unreliable Results, Unreliable Data, Results Not Reproducible, Original Data not Provided, Manipulation of Results, Manipulation of Images, Falsification/Fabrication of Results, Falsification/Fabrication of Data, Error in Data, Concerns/Issues About Results, Concerns/Issues About Image, Concerns/Issues About Data, Unreliable Image, Falsification/Fabrication of Data, Falsification/Fabrication of Image |
| Duplicate Publication | Withdrawn to Publish in Different Journal, Taken from Dissertation/Thesis, Salami Slicing, Euphemisms for Duplication, Duplication of Text, Duplication of Data, Duplication of Article, Duplicate Publication through Error by Journal/Publisher, Duplication of Image |
| Error / Mistake | Error in Text, Error in Results and/or Conclusions, Error in Methods, Error in Materials (General), Error in Image, Error in Cell, Lines/Tissues, Error in Analyses, Error by Third Party, Error by Journal/Publisher, Contamination of Reagents, Contamination of Materials (General), Contamination of Cell Lines/Tissues |
| Authorship Dispute | Objections by Author(s), FALSE/Forged Authorship, Concerns/Issues About Authorship, FALSE/Forged Authorship, Concerns/Issue, About Authorship, Lack of Approval from Author, False/Forged Authorship |
| Fake Review Process | Fake Peer Review, Concerns/Issues with Peer Review |
| Copyright Issues | Copyright Claims, Conflict of Interest |
| Others | Concerns/Issues about Referencing/Attributions, Paper Mill, Randomly Generated Content,, Author Unresponsive, Lack of IRB/IACUC Approval, Concerns/Issues About Referencing/Attributions, Concerns/Issues about Human Subject Welfare, Informed/Patient Consent - None/Withdrawn, Objections by Third Party, Ethical Violations by Author, Concerns/Issues About Third Party Involvement, Misconduct by Author, Lack of Approval from Third Party, Breach of Policy by Author, Lack of Approval from the Company/Institution, Misconduct-Official Investigation/Finding, Retract and Replace, Publishing Ban, Ethical Violations by Third Party, False Affiliation, Misconduct by Third Party, Cites Retracted Work, Legal Reasons/Legal Threats, Bias Issues or Lack of Balance, Complaints about Author, Miscommunication by Author, Complaints about Third Party, Concerns/Issues about Animal Welfare, Objections by Company/Institution, Civil Proceedings, Breach of Policy by Third Party, Taken via Peer Review, No Further Action, Miscommunication by Third Party, Temporary Removal, Nonpayment of Fees/Refusal to Pay, Misconduct by Company/Institution, Miscommunication by Journal/Publisher, Euphemisms for Misconduct, Misconduct-Official Investigation/Finding |
| Unknown | Investigation by Journal/Publisher, Investigation by Third Party, Rogue Editor, Date of Retraction/Other Unknown, Investigation by Company/Institution, Notice - Limited or No Information, Upgrade/Update of Prior Notice, Withdrawal, Notice-Limited or No Information, Notice - No/Limited Information, Updated to Retraction, Doing the Right Thing Withdrawn (out of date),Notice - Unable to Access via current resources, Notice-Lack of, Updated to Correction, Investigation by ORI, Notice-Unable to Access via current resources |
