# Supplementary material for: Mapping retracted articles and exploring regional differences in China, 2012–2023
Source: PLoS One. 2024 Dec 2;19(12):e0314622. doi: 10.1371/journal.pone.0314622 (PMC11611127; doi:10.1371/journal.pone.0314622)
Supplement: S6 Table — (DOCX) [file pone.0314622.s006.docx]

**S6 Table. The reasons for retractions**

|  | **2012** | **2013** | **2014** | **2015** | **2016** | **2017** | **2018** | **2019** | **2020** | **2021** | **2022** | **2023** | **2012-2023** |
| --- | --- | --- | --- | --- | --- | --- | --- | --- | --- | --- | --- | --- | --- |
| Plagiarism | 7.91% | 8.22% | 17.30% | 10.32% | 16.07% | 10.05% | 10.77% | 12.24% | 3.77% | 1.32% | 1.45% | 0.71% | 2.01% |
| Fake Data | 22.09% | 7.05% | 17.65% | 9.66% | 13.79% | 15.93% | 22.07% | 23.00% | 27.27% | 21.54% | 22.23% | 32.06% | 29.14% |
| Duplicate Publication | 10.00% | 11.15% | 14.88% | 9.66% | 11.51% | 9.19% | 12.23% | 10.76% | 8.58% | 9.65% | 7.39% | 1.99% | 4.87% |
| Error / Mistake | 4.42% | 4.31% | 13.15% | 7.41% | 10.07% | 7.84% | 17.82% | 12.24% | 6.89% | 3.42% | 2.63% | 0.83% | 2.53% |
| Authorship Dispute | 1.16% | 1.57% | 2.08% | 2.65% | 2.28% | 3.92% | 3.72% | 4.17% | 2.20% | 1.01% | 2.09% | 0.90% | 1.35% |
| Fake Review Process | 1.40% | 0.98% | 2.08% | 17.06% | 9.95% | 17.52% | 1.99% | 4.17% | 1.26% | 6.52% | 16.69% | 12.00% | 11.18% |
| Copyright Issues | 1.40% | 1.57% | 1.38% | 1.06% | 0.60% | 1.23% | 2.79% | 1.04% | 0.44% | 0.27% | 0.23% | 0.06% | 0.24% |
| Others | 7.91% | 7.44% | 11.76% | 13.23% | 8.03% | 7.72% | 8.91% | 14.41% | 19.98% | 23.83% | 16.83% | 25.24% | 22.19% |
| Unknown | 43.72% | 57.73% | 19.72% | 28.70% | 26.98% | 25.98% | 19.15% | 17.01% | 23.78% | 29.91% | 27.91% | 25.43% | 26.49% |

Note: The figure represents the percentage of reason for retraction.
