# Supplementary material for: Mapping retracted articles and exploring regional differences in China, 2012–2023
Source: PLoS One. 2024 Dec 2;19(12):e0314622. doi: 10.1371/journal.pone.0314622 (PMC11611127; doi:10.1371/journal.pone.0314622)
Supplement: S7 Table — (DOCX) [file pone.0314622.s007.docx]

**S7 Table. The top3 Classification of other reasons for retraction**

| **Others** | **2012** | **2013** | **2014** | **2015** | **2016** | **2017** | **2018** | **2019** | **2020** | **2021** | **2022** | **2023** | **2012-2023** |
| --- | --- | --- | --- | --- | --- | --- | --- | --- | --- | --- | --- | --- | --- |
| Concerns/Issues about Referencing/Attributions | 0.00% | 0.00% | 0.00% | 0.00% | 0.00% | 0.00% | 0.00% | 0.00% | 0.00% | 0.00% | 0.00% | 10.55% | 6.18% |
| Paper Mill | 0.00% | 0.00% | 0.00% | 0.00% | 0.12% | 0.35% | 0.13% | 0.35% | 7.87% | 9.66% | 9.37% | 3.62% | 5.16% |
| Randomly Generated Content | 1.16% | 0.39% | 0.35% | 1.19% | 0.00% | 0.00% | 0.00% | 0.09% | 1.29% | 5.58% | 0.03% | 5.48% | 3.99% |

Note: The figure represents the percentage of reason for retraction.
