# Supplementary figures and images for: Mapping retracted articles and exploring regional differences in China, 2012–2023
Source: PLoS One. 2024 Dec 2;19(12):e0314622. doi: 10.1371/journal.pone.0314622 (PMC11611127; doi:10.1371/journal.pone.0314622)

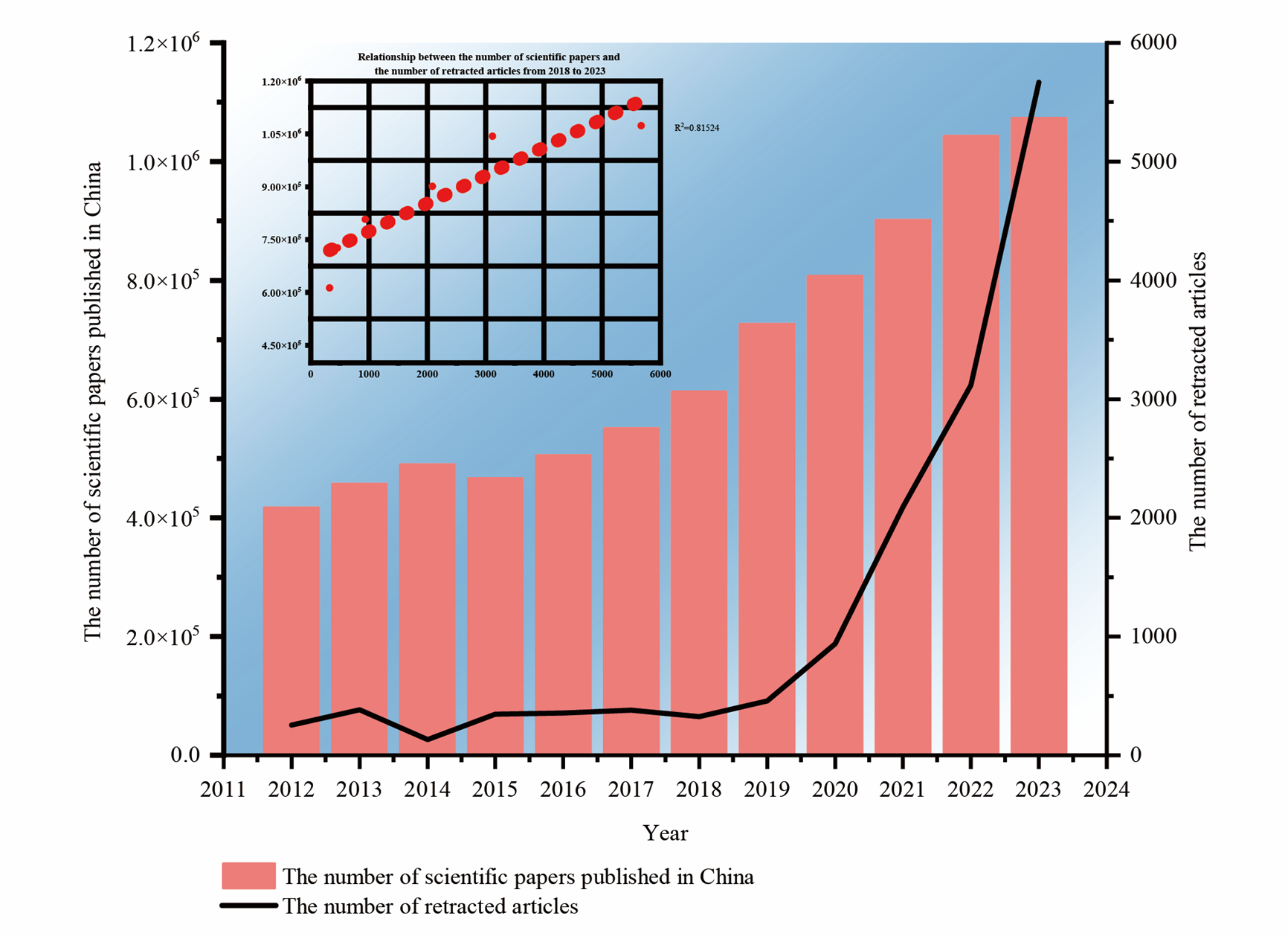

Supplement: S1 Fig — Note: The number of publications with Chinese authors is based on data from Scopus, retrieved August 29, 2024. (TIF) [file pone.0314622.s008.tif]

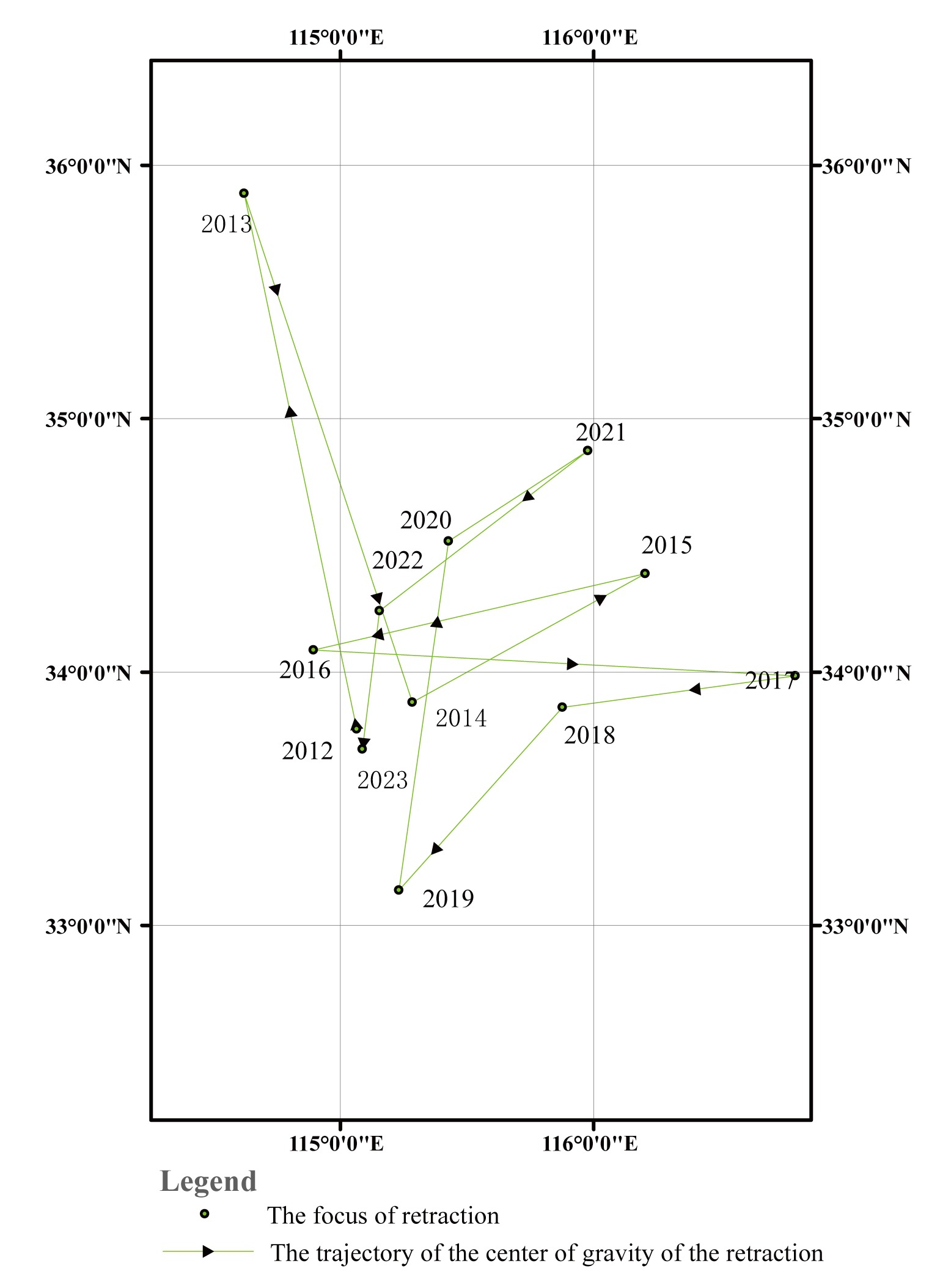

Supplement: S2 Fig — (TIF) [file pone.0314622.s009.tif]
