# Supplementary material for: Mapping retracted articles and exploring regional differences in China, 2012–2023
Source: PLoS One. 2024 Dec 2;19(12):e0314622. doi: 10.1371/journal.pone.0314622 (PMC11611127; doi:10.1371/journal.pone.0314622)
Supplement: Attachment — Submitted filename: Review Report.docx [file pone.0314622.s010.docx]

I appreciate the authors for conducting a study of retracted articles written by Chinese authors using data from the Retraction Watch database. This study focuses on retracted articles written by Chinese authors from 31 provinces in 25 mainland China during the study period between January 1, 2012, and December 31, 2023. The author may consider explaining the following points:

The author conducted a subject-wise analysis of retracted articles and the reasons behind them, focusing on Chinese authors from 31 provinces over a 12-year period. Does this analysis alone suffice to justify the significance of retracted articles and the exploration of regional differences in China?

It lacks institutes, publishers, and authors in particular; it should have at the very least list the top 10 of these.

The authors used the words “retracted article” and “retracted manuscript” in most places. For example, on **page 9, line 179**, the author writes about the subject analysis of retracted manuscripts in China, and on **page 9, line 180**, the author writes about the subject analysis of retracted articles from Chinese authors. The research is based on retracted articles, not manuscripts.

**Figure 4**: Autocorrelation analysis mapping is difficult to understand; you can provide a table or provide more explanations.

**Supplement Information Fig. S1, Page 7, Line 21 and 22:** Authors has mentioned data source of the research is from Retraction Watch database for retracted articles and the number of publications with Chinese authors is based on data from the Web of Science. Scopus and Web of Science are the two major databases, Scopus covers more peer-reviewed journals than Web of Science, my question is why authors chose Web of Science for year wise publication.

**Supplement Information Fig. S1, Page 7, Lines 21 and 22**: The authors have mentioned that the data source for the research is from Retraction Watch database for retracted articles, and the number of publications with Chinese authors is based on data from the Web of Science. Scopus and Web of Science are the two major databases. Scopus covers more peer-reviewed journals than Web of Science. My question is why authors chose Web of Science for year-wise publication.

Overall, I suggest the author may include additional up-to-date references on retracted articles.
