# Supplementary material for: Mapping retracted articles and exploring regional differences in China, 2012–2023
Source: PLoS One. 2024 Dec 2;19(12):e0314622. doi: 10.1371/journal.pone.0314622 (PMC11611127; doi:10.1371/journal.pone.0314622)
Supplement: Attachment — Submitted filename: Response to Reviewers.docx [file pone.0314622.s011.docx]

**Response to Reviewers**

**Dear Editor,**

We are grateful for the valuable comments and suggestions given by the reviewers concerning our manuscript entitled “**Mapping retracted articles and exploring regional differences in China, 2012-2023**” **(ID: PONE-D-24-27000).** We give our responses to the reviewer in this letter. We hope that after our replies, our manuscript will be considered for publication in **PLOS ONE**. The detailed responses are listed below, and revised parts of the manuscript are marked with red color.

**Question 1. Figures 3, 5, and 6 in your submission contain [map/satellite] images which may be copyrighted**

**Response:** Thanks. We are sorry that we are unable to obtain permission from the original copyright holder to publish Figures 3, 5, and 6 under the CC BY 4.0 license. We have learned that the map images we utilized must be submitted for review to the com-petent geospatial information administration department, which holds the authority for map review" (https://www.gov.cn/zhengce/2015-12/14/content_5023591.htm). Moreover, map review applications can only be submitted by legal entities, not by individuals, thus we have revised these figures as follows:

Fig 3 has been replaced with a bubble map plotted using Origin pro 2024b (Origin Lab Co., Northampton, MA, USA, Serial number DL2W8-6089-7609063).

Fig 5 and Fig 6 have beeb replaced with Table 2 and Table 3 to present the results of the Local spatial autocorrelation analysis and Getis-Ord Gi * analysis.

We have checked copyright information on all replacement figures and updated the figure caption with source information.

**Fig 3. Spatial distribution of the number of retracted articles and the number of the major research institutes across China’s 31 provinces from 2012 to 2023.**

Note: According to the definitions provided by the National Bureau of Statistics, all provinces were categorized into four regions: the East, the West, the Central and the North-east. The point of interest (POI) datasets regarding the number of major research institutions, including both research institutes and institutions of higher learning, were obtained from Google Maps in June 2023.

| **Table 2. Local spatial autocorrelation analysis of the retraction counts in the 31 provinces of China** | | | | |
| --- | --- | --- | --- | --- |
| **Year** | **Type of Clustering** | | | |
|  | **H-H clustering** | **H-L clustering** | **L-H clustering** | **L-L clustering** |
| 2017 | Jiangsu | Shaanxi |  | Xinjiang Uygur Autonomous Region, Tibet Autonomous Region, Sichuan, Yunnan, Gansu, and Qinghai |
| 2018 | Jiangsu, Shanghai |  | Anhui | Xinjiang Uygur Autonomous Region, Gansu, Sichuan, and Yunnan |
| 2019 | Jiangsu, Shanghai | Sichuan, Liaoning | Anhui | Xinjiang Uygur Autonomous Region and Inner Mongolia Autonomous Region |
| 2021 |  | Sichuan | Anhui | Xinjiang Uygur Autonomous, Qinghai, Gansu, and Yunnan |
| 2023 | Henan, Jiangsu, Shandong, Anhui, and Shanghai | Sichuan |  | Xinjiang Uygur Autonomous Region, Qinghai, Tibet Autonomous Region, and Gansu |
| 2012-2023 | Jiangsu, Shandong | Sichuan | Anhui | Xinjiang Uygur Autonomous, Qinghai, and Gansu |

| **Table 3. Getis-Ord Gi * analysis results of retracted articles from 31 provinces in China** | | | | | | |
| --- | --- | --- | --- | --- | --- | --- |
| **Type of Clustering** | **Year** | | | | | |
|  | **2017** | **2018** | **2019** | **2021** | **2023** | **2012-2023** |
| Hot spot 99% CI | Jiangsu, Shanghai | Jiangsu, Shanghai | Jiangsu, Shanghai,  Anhui | Shandong | Shandong, Jiangsu, Anhui | Shandong, Jiangsu, Anhui |
| Hot spot 95% CI |  | Anhui | Shandong | Jiangsu, Anhui, Henan | Henan, Shanghai | Henan, Shanghai |
| Hot spot 90% CI |  | Zhejiang | Zhejiang |  |  |  |
| Cold spot 99% CI |  |  |  |  | Xinjiang Uygur  Autonomous Region |  |
| Cold spot 95% CI |  |  |  |  | Tibet Autonomous Region, Qinghai | Xinjiang Uygur  Autonomous Region, Qinghai |
| Cold spot 90% CI | Xinjiang Uygur Autonomous Region, Tibet Autonomous Region, Sichuan, Gansu, Qinghai | Xinjiang Uygur Autonomous Region, Gansu,  Sichuan, Yunnan | Inner Mongolia Autonomous Region | Xinjiang Uygur Autonomous Region, Tibet Autonomous Region, Sichuan, Qinghai | Gansu, Sichuan,  Yunnan | Tibet Autonomous Region, Gansu,  Sichuan, Yunnan |

**Question 2. a) Please provide search terms that can be used on the Retraction Watch Database to access the original dataset(s).**

**b.) Please clarify if you have permission from the original database to reupload their data to Figshare.**

**Response:** Thanks.

After signing a data use agreement, we downloaded the dataset from the Retraction Watch Database on July 25, 2022 (https://api.labs.crossref.org/data/retractionwatch?name@email.org (add your ‘mailto’)) and updated the data for 2022 and 2023 on May 16, 2024. The usage agreement for the Retraction Watch Database is shown in Fig S3. The Retraction Watch Database was completely open and freely available on September 12, 2023.

The records downloaded from Retraction Watch Database were filtered by the country “China” and included only those with a retraction date between January 1, 2012, and December 31, 2023. This study focuses solely on the institutions of the first authors of the retracted articles from the 31 provinces in mainland China. The details of the provinces were extracted from the “institution” field within the Retraction Watch Database. A total of 14,445 retraction records were ultimately included in our study. The selection process of retracted articles is detailed in Fig 1.

Additionally, we have reuploaded this data to Figshare. https://doi.org/10.6084/m9.figshare.27044899.

**Fig S3. The usage agreement for Retraction Watch Database**

**Fig 1. The selection process of retracted articles from the Retraction Watch Database**

**Question 3: Please upload the minimal data set necessary to replicate your study's findings to a stable, public repository (such as Figshare or Dryad) and provide us with the relevant URLs, DOIs, or accession numbers that may be used to access these data.**

**Response:** Thanks. We have uploaded the necessary minimum dataset to Figshare ([https://](https://doi.org/10.6084/m9.figshare.26395126.v1) /doi.org/10.6084/m9.figshare.27044899).

**Question 4: Please include captions for your Supporting Information files at the end of your manuscript, and update any in-text citations to match accordingly.**

**Response:** Thanks. We have added captions for the Supporting Information files at the end of the revised manuscript and updated any in-text citations to match accordingly in our revised manuscript.

**Reviewer #1:**

**Question 1: (pg. 9, ln. 57) cite more literature. In many studies****, China tops the list of retractions.**

**Response:** Thanks for your suggestion. We have cited more literature in the revised manuscript to support the statement that China tops the list of retractions

The literatures were cited as follows:

Van Noorden R. More than 10,000 research papers were retracted in 2023-a new record. Nature 2023;624(7992):479-481.<https://doi.org/10.1038/d41586-023-03974-81>.

Zhang Q, Abraham J, Fu H. Collaboration and its influence on retraction based on retracted publications during 1978–2017. Scientometrics 2020; 125:213-232. https://doi.org/10.1007/s11192-020-03636-w.

Ataie-Ashtiani B. Chinese and Iranian Scientific Publications: Fast Growth and Poor Ethics. Sci Eng Ethics 2017;23(1):317-319. https://doi.org/10.1007/s11948-016-9766-1.

Xiao Y, Chen J, Wu XH, Qiu QM. High retraction rate of Chinese articles: it is time to do something about academic misconduct. Postgrad Med J 2022;98(1163):653-654. https://doi:10.1136/postgradmedj-2021-140853. PMID: 37062972.

Eldakar MAM, Shehata AMK. A bibliometric study of article retractions in technology fields in developing economies countries. Scientometrics 2023;128(11):6047-6083. https://doi.org/10.1007/s11192-023-04823-1.

**Question 2: (pg. 11, ln. 87) Provide retraction watch database website link**

**Response:** Thanks for your suggestion. We have added the **Retraction Watch Database website link** on page 5, line 93 in the revised manuscripts, as follows:

We utilized the Retraction Watch Database for our study (http://www.retractiondatabase.org/RetractionSearch.aspx).

**Question 3: (pg. 11, ln. 97) cite studies conducted using retraction watch database**

**Response:** Thanks for your suggestion. We have cited studies conducted using the Retraction Watch Database in the revised manuscript, as follows:

Yang W, Sun N, Song H. Analysis of the retraction papers in oncology field from Chinese scholars from 2013 to 2022. J Cancer Res Ther 2024;20(2):592-598. https://doi:10.4103/jcrt.jcrt_1627_23. Epub 2024 Apr 30. PMID: 38687929.

Kocyigit BF, Zhaksylyk A, Akyol A, Yessirkepov M. Characteristics of Retracted Publications From Kazakhstan: An Analysis Using the Retraction Watch Database. J Korean Med Sci 2023;38(46): e390. https://doi:10.3346/jkms.2023.38. e390. PMID: 38013646; PMCID: PMC10681843.

Zhong QY, Zhang XY, Luo HH, Jiang X, Zeng XY, Jiang J, et al. [Analysis of the characteristics of retracted scientific papers in the field of global liver diseases published by Chinese scholars]. Zhonghua Gan Zang Bing Za Zhi 2023;31(1):96-100. https://doi:10.3760/cma.j.cn501113-20210324-00138. PMID: 36948856.

.

**Question 4: (pg. 13, ln. 138) cites literature that used similar tests to substantiate the analysis.**

**Response:** Thanks for your suggestion. We have cited literature that used similar tests to substantiate the analysis in the revised manuscripts as follows:

Sako S, Gilano G, Dileba T, Ayenew T, Addis Y. Spatial distribution and determinants of exclusive breastfeeding practice among mothers of children under 24 months of age in Ethiopia: spatial and multi-level analysis. BMC Pregnancy Childbirth 2024;24(1):554.

**Question 5: (pg. 14, ln. 153) What could be the reason(s) for a sharp surge in retractions within China’s scientific community, with an alarming peak of 5,668 retractions in 2023? Justify.**

**Response:** Thanks for your suggestion. The sharp increase in retractions within China's scientific community, which reached an alarming peak of 5,668 retractions in 2023, may be attributed to multiple factors and we have supplemented in the section of discussion in our revised manuscript. The factors are as follows:

Primarily, the number of retracted articles showed a substantial correlation with the volume of articles published(36). In our study, we found that the number of retracted articles was significantly correlated with the volume of articles published in the years 2018–2023. Notably, the number of Chinese publications reached 1,073,392 in Scopus in 2023. Furthermore, since 1990, China has implemented a heightened level of research funding and incentives for researchers(37). The increase in the number of retracted articles may also be a consequence of the “publish or perish” culture, rewarding quantity over quality in academic productivity to attain graduation, promotion, and rewards, which may lead to academic misconduct and eventually to an increased frequency of retractions (38). Innovation in monitoring and reporting research misconduct, including extensive testing of Retraction Warch Databases(39), as well as increasing scrutiny from China and publishers(such as Hindawi) (4, 16), and the widespread use of plagiarism detection software(40-41), may contribute to the detection and reporting of a greater number of retracted articles.

**Question 6: (pg. 15, ln. 161) “The retraction rate rose sharply after 2019 and reached 0.64% in 2023” Reason (s) for a sharp increase in the retraction rate?**

**Response:** Thanks for your suggestion. The sharp increase in the retraction rate can be attributed to several key factors, which we have supplemented in the section of discussion in our revised manuscript. As follows:

There is one other point worth noting our study showed that the retraction rate rose sharply after 2019 and reached 0.53% in 2023. Above we have analyzed why the peak in retractions in 2023 is associated with increased research output, “publish or perish” culture, and advancements in detecting and addressing research misconduct. In addition, the growth rate of retracted articles outpaced the growth rate of published papers during 2019–2023 compared to 2012-2018. These may be the reasons for a sharp increase in the retraction rate.

**Question 7: Suggesting that authors incorporate broader reflection on the implications of your findings for the field as a whole is needed and the concluding sentences should come up with some reflections regarding your takeaways from the study.**

**Response:** Thanks for your suggestion. We have incorporated broader reflection on the implication of our findings for the field as a whole in the section of discussion in the revised manuscript and modified the conclusion as follows:

This study mainly attempts to remedy the current lack of research about the spatial distribution of retracted articles by Chinese authors. Notably, there is a discernible spatial clustering among these retractions, with a gradual increase in the number of retracted articles from west to east. Shandong, Jiangsu, Shanghai, Henan, and Anhui are the hotspots for retractions. In view of this, the causes of retraction should be further analyzed for these highly aggregated areas within the context of each province in order to take more precise prevention and control measures.

**Question 8: Although the subject of the study is worthy of further exploration, the current paper does not offer new insights that address research gaps or provide practical implications for policymakers, institutions, and academicians.**

**Response:** Thanks for your suggestion. We have revised the section of the discussion in the Revised Manuscript to provide practical implications for policymakers, institutions, and academicians. As following:

Therefore, actively implementing measures to reduce the emphasis on publications in professional title evaluations and other utilitarian requirements may significantly decrease the incidence of retractions. Furthermore, enhancing the rigorous review and verification of article data prior to publication could serve as a strong deterrent against data fabrication and related issues. Additionally, it is essential to strengthen punitive measures and promote education on research integrity.

**Question 9: There is inconsistency in in-text citations. For example (pg. 9, ln 42) is cited as “an aspect of science (5)”; (pg. 16, ln 192) is cited as “distinct categories (24)” (pg. 9, ln 44) is cited as “eliminate the scientific environment (6, 7)” (pg. 9, ln 57) is cited as “retracted publications (13) (14)” Suggesting author(s) check and follow the authorship guidelines.**

**Response:** Thanks. We have thoroughly reviewed the manuscript to ensure that all in-text citations are consistent and correctly formatted according to the authorship guidelines.

**Reviewer #2**

**Question 1: The author conducted a subject-wise analysis of retracted articles and the reasons behind them, focusing on Chinese authors from 31 provinces over 12 years. Does this analysis alone suffice to justify the significance of retracted articles and the exploration of regional differences in China? It lacks** **institutes, publishers, and authors in particular; it should have at the very least listed the top 10 of these.**

**Response:** Thanks for your reasonable proposal. We have added an analysis of institutes, publishers, and authors in particular and listed the top 10 of these in the revised manuscript as follows:

A total of 14,445 retracted articles were published by 203 publishers from 2012 to 2023. The top 10 publishers involved in the retractions are presented: Hindawi (30.90%), Springer(14.57%), Elsevier(9.74%), Taylor and Francis(5.24%), Wiley(4.78%), IOP Publishing(3.99%), Spandidos(3.16%), SAGE Publications(3.07%), IEEE (2.74%), Association for Computing Machinery (ACM) (2.35%). The retractions involved 6466 journals during 2012 -2023, the top 10 journals with the highest number of retractions include China-Japan Union Hospital of Jilin University, The First Hospital of Jilin University, The First Affiliated Hospital of Zhengzhou University, Central South University, and so on. Please refer to the S3 and S4 Tables for specific details.

**S3Table. The top 10 publishers involved retracted articles from Chinese authors published between 2012 and 2023**

| **Publishers** | **Number of retracted articles** | **Proportion** |
| --- | --- | --- |
| Hindawi | 4464 | 30.90% |
| Springer | 2105 | 14.57% |
| Elsevier | 1407 | 9.74% |
| Taylor and Francis | 757 | 5.24% |
| Wiley | 691 | 4.78% |
| IOP Publishing | 577 | 3.99% |
| Spandidos | 457 | 3.16% |
| SAGE Publications | 444 | 3.07% |
| IEEE | 396 | 2.74% |
| Association for Computing Machinery (ACM) | 339 | 2.35% |

**S4 Table. The top 10 institutions involved retracted articles from Chinese first authors**

**institutions between 2012 and 2023**

| **Institutions*** | **Number of retracted**  **articles** | **Proportion** |
| --- | --- | --- |
| China-Japan Union Hospital of Jilin University | 152 | 1.05% |
| The First Hospital of Jilin University | 90 | 0.62% |
| The First Affiliated Hospital of Zhengzhou University | 85 | 0.59% |
| Central South University | 64 | 0.44% |
| The Second Hospital of Jilin University | 50 | 0.35% |
| Jilin University | 49 | 0.34% |
| The Affiliated Hospital of Qingdao University | 48 | 0.33% |
| Zhejiang University | 46 | 0.32% |
| Sichuan University | 44 | 0.30% |
| The University of Electronic Science and Technology of China | 44 | 0.30% |

*The affiliation of the first author.

**Question 3:** **The authors used the words “retracted article” and “retracted manuscript” in most places. For example, on page 9, line 179, the author writes about the subject analysis of retracted manuscripts in China, and on page 9, line 180, the author writes about the subject analysis of retracted articles from Chinese authors. The research is based on retracted articles, not** **manuscripts.**

**Response:** Thank you very much. We have replaced the term “Retracted manuscripts” with “Retracted article” and have thoroughly verified this correction in the revised manuscript.

**Question 4:** **Figure 4: Autocorrelation analysis mapping is difficult to understand; you can provide a table or provide more explanations.**

**Response:** Thank you very much for your suggestions. We have added mote explanations about fig 4 as follows:

Fig 4 is the result of trend surface analysis. The trend surface analysis is a multivariate statistical method based on the least squares approach, and this method was employed to accurately fit the regional distribution and temporal trends in sample data. By considering the number of retracted articles with Chinese authors across 31 provinces along with the corresponding longitude or latitude of each province, we established a binary polynomial regression model and presented it through a three-dimensional visualization to effectively demonstrate the overall spatial trend of retraction occurrences. In this study, we used ArcGIS 10.3 to analyze, the result shown in:

Unfortunately, the results can only be presented in the form of graphs, and the software cannot output the corresponding value. The X-axis and Y-axis represent longitude and latitude, and the Z-axis represents the number of retracted papers in each year. A point (X, Y, Z) in the three-dimensional space represents the number of retracted papers in a certain research area. The green line represents the east-west direction, and the blue line represents the north-south direction. The steeper the trend line change, the greater the difference.

The results described in Fig4 was presented in the section of results as follows:

The trend surface fitting results indicate that the spatial trend of 31 provinces in China demonstrates an inverted ‘U’ type distribution in the north-south direction from 2012 to 2023. Moreover, there is a noteworthy spatial directivity pattern in the east-west direction, with elevated values in the east and lower values in the west. For instance, in 2023, as longitude increases, the number of retractions also increases; meanwhile, as dimension decreases, the number of retractions initially rises and then declines. High-heat areas for the number of retractions are identified in the eastern and central regions. In addition, the trend line alterations vary in differing directions, with a steeper transition of the trend surface in the east-west direction and a comparatively smoother transition in the north-south direction. Therefore, the difference in the number of retractions is more pronounced in the east-west direction, as depicted in Fig 4.

**Question 5: Supplement Information Fig. S1, Page 7, Lines 21 and 22: Authors have mentioned data source of the research is from** **the Retraction Watch database for retracted articles and the number of publications with Chinese authors is based on data from the Web of Science. Scopus and Web of Science are the two major databases, Scopus covers more peer-reviewed journals than Web of Science, my question is why authors chose Web of Science for year-wise publication.**

**Response:** Thank you very much. We apologize for previously overlooking the fact that Scopus encompasses a broader range of publications than the Web of Science; consequently, we have supplemented our data obtained from Scopus in S1 Table.

**S1 Table.** **The number of publications (Retraction rate) by Chinese authors from 2012-2023 based on Scopus and Web of Science**

| **Year** | **The number of publications (Retraction rate)** | |
| --- | --- | --- |
|  | **Web of Science** | **Scopus** |
| 2012 | 202832（0.12%） | 417,455（0.06%） |
| 2013 | 240900（0.05%） | 457,394（0.08%） |
| 2014 | 276009（0.11%） | 489,452（0.03%） |
| 2015 | 308850（0.10%） | 466,722（0.07%） |
| 2016 | 341298（0.10%） | 505,047（0.07%） |
| 2017 | 377792（0.10%） | 551,119（0.07%） |
| 2018 | 436305（0.07%） | 612,763（0.05%） |
| 2019 | 536132（0.09%） | 726,245（0.06%） |
| 2020 | 616085（0.15%） | 807,322（0.12%） |
| 2021 | 727457（0.29%） | 901,191（0.23%） |
| 2022 | 883953（0.35%） | 1,043,351（0.30%） |
| 2023 | 885338（0.64%） | 1,073,392（0.53%） |

**Fig 2 The trend of retraction rate in China over time.**

Note：The number of publications with Chinese authors each year was obtained from the web of science (A) and Scopus (B).

**Question 6:** **Overall, I suggest the author include additional up-to-date references on retracted articles.**

**Response:** Thank you very much for your suggestions. We have included additional latest references on retracted articles.

**Reviewer #3**

**Question 1: In page 9, a sentence reads "No province had more than 106 retractions before 2020" which is not clear.**

**Response:** Thanks for your reasonable proposal. We have revised the sentence as follows:

Before 2020, the number of retracted articles from each province in China was fewer than 114

**Question 2: On page 9, there is note below Figure 2, "Note: The number of publications with Chinese authors each year was obtained from the Web of Science" which is contradictory to the data source mentioned in the Methodology section.**

**Response:** Thank you for your feedback. We have revised the Methodology section for clarity in revised manuscript.

The percentage of retractions by year was calculated by using the number of original articles published and subsequently retracted in given year as the numerator, and the total number of publications in the same year as the denominator.

The annual number of retractions by Chinese authors was derived from the Retraction Watch database, which is the most comprehensive index of retractions, covering articles published from 1753 to 2020. Retraction records are collected daily through various sources, including email alerts, user submissions, journal contents, PubMed, Google Scholar, and publisher sites such as SpringerLink and ScienceDirect.

We utilized the Web of Science to acquire annual publication numbers. Given that Scopus covers a broader range of peer-reviewed journals, we have supplemented data from Scopus to acquire the annual publication numbers and updated the results section in the revised manuscript.

**Question 5: In page 10, an in-text citation "distinct categories24" which is not formatted and the provided reference is not appropriate. Please check the reference provide.**

**Response:** Thanks for your suggestion. We have checked the reference as follows:

Elango B. Retracted articles in the biomedical literature from Indian authors. Scientometrics 2021;126(5):3965-3981. https://doi:10.1007/s11192-021-03895-1. Epub 2021 Mar 7. PMID: 33716353.

**Question 3: Subject analysis needs more details.**

**Response:** Thank you for your suggestion. We have added more details about subject analysis in revised manuscript.

The subject classification used in this paper is taken from the Retraction Watch Database, mainly encompassing the following fields: Business & Technology, Environmental Sciences, Health Sciences, Physical Sciences, Basic Life Sciences, Social Sciences, and Humanities. The official guide is available at the following link: <https://retractionwatch.com/retraction-watch-database-user-guide/retraction-watch-database-user-guide-appendix-a-fields/>.

A retracted article may have one or more retractions subject, each of which is counted separately, for a subject of 41,657. Analysis of the subject of retracted articles from Chinese authors between 2012 and 2023 reveals that retraction was most common in basic life sciences (40.66%), health sciences (21.46%), business and technology (20.32%), with lower percentages in physical sciences (7.12%), environmental sciences (1.81%), social sciences (7.37%), and humanities (1.25%). The basic life sciences are mainly focused on Cellular (27.38%), Genetics (21.46%) and Cancer (19.93%). As shown in S2 Table.

**S2** **Table The distribution of subject of retracted articles from Chinese authors between 2012 and 2023(N/%)**

| **Subject** | | **2012** | **2013** | **2014** | **2015** | **2016** | **2017** | **2018** | **2019** | **2020** | **2021** | **2022** | **2023** | **2012-2023** |
| --- | --- | --- | --- | --- | --- | --- | --- | --- | --- | --- | --- | --- | --- | --- |
| Physical Sciences (PHY) | total | 204/43.13% | 210/34.88% | 51/14.57% | 108/10.96% | 129/11.91% | 139/9.69% | 187/21.62% | 187/14.56% | 219/6.87% | 327/5.54% | 350/4.12% | 856/5.03% | 2967/7.12% |
|  | Astronomy | 0/0.00% | 0/0.00% | 0/0.00% | 0/0.00% | 0/0.00% | 0/0.00% | 0/0.00% | 0/0.00% | 0/0.00% | 0/0.00% | 0/0.00% | 1/0.12% | 1/0.04% |
|  | Astrophysics | 0/0.00% | 0/0.00% | 0/0.00% | 0/0.00% | 1/0.78% | 1/0.72% | 2/1.07% | 2/1.07% | 0/0.00% | 1/0.31% | 2/0.57% | 1/0.12% | 10/0.37% |
|  | Chemistry | 46/22.55% | 14/6.67% | 14/27.45% | 20/18.52% | 31/24.03% | 26/18.71% | 27/14.44% | 43/22.99% | 34/15.53% | 39/11.93% | 27/7.71% | 34/3.97% | 355/13.16% |
|  | Cosmology | 0/0.00% | 0/0.00% | 0/0.00% | 0/0.00% | 0/0.00% | 0/0.00% | 0/0.00% | 0/0.00% | 1/0.46% | 0/0.00% | 1/0.29% | 0/0.00% | 2/0.07% |
|  | Crystallography/Spectroscopy | 30/14.71% | 3/1.43% | 2/3.92% | 3/2.78% | 1/0.78% | 4/2.88% | 16/8.56% | 10/5.35% | 15/6.85% | 16/4.89% | 6/1.71% | 7/0.82% | 113/4.19% |
|  | Energy | 11/5.39% | 5/2.38% | 0/0.00% | 3/2.78% | 0/0.00% | 3/2.16% | 14/7.49% | 11/5.88% | 3/1.37% | 13/3.98% | 25/7.14% | 114/13.32% | 202/7.49% |
|  | Engineering –  Chemical | 7/3.43% | 8/3.81% | 2/3.92% | 10/9.26% | 9/6.98% | 18/12.95% | 18/9.63% | 18/9.63% | 28/12.79% | 27/8.26% | 30/8.57% | 35/4.09% | 210/7.79% |
|  | Engineering –  Electrical | 22/10.78% | 10/4.76% | 0/0.00% | 10/9.26% | 10/7.75% | 14/10.07% | 25/13.37% | 20/10.70% | 14/6.39% | 22/6.73% | 39/11.14% | 93/10.86% | 279/10.34% |
|  | Engineering –  General | 44/21.57% | 107/50.95% | 0/0.00% | 3/2.78% | 3/2.33% | 5/3.60% | 2/1.07% | 1/0.53% | 1/0.46% | 37/11.31% | 25/7.14% | 83/9.70% | 311/11.53% |
|  | Engineering  Mechanical | 6/2.94% | 9/4.29% | 2/3.92% | 4/3.70% | 7/5.43% | 1/0.72% | 12/6.42% | 9/4.81% | 3/1.37% | 13/3.98% | 20/5.71% | 52/6.07% | 138/5.12% |
|  | Engineering –  Structural | 3/1.47% | 4/1.90% | 2/3.92% | 3/2.78% | 3/2.33% | 8/5.76% | 6/3.21% | 5/2.67% | 10/4.57% | 14/4.28% | 21/6.00% | 50/5.84% | 129/4.78% |
|  | Geology | 1/0.49% | 5/2.38% | 3/5.88% | 8/7.41% | 5/3.88% | 4/2.88% | 2/1.07% | 5/2.67% | 13/5.94% | 16/4.89% | 9/2.57% | 22/2.57% | 93/3.45% |
|  | Hydrology | 4/1.96% | 4/1.90% | 0/0.00% | 2/1.85% | 1/0.78% | 0/0.00% | 2/1.07% | 1/0.53% | 1/0.46% | 1/0.31% | 2/0.57% | 1/0.12% | 19/0.70% |
|  | Materials Science | 12/5.88% | 18/8.57% | 11/21.57% | 19/17.59% | 27/20.93% | 31/22.30% | 20/10.70% | 20/10.70% | 27/12.33% | 34/10.40% | 45/12.86% | 160/18.69% | 424/15.72% |
|  | Mathematics | 6/2.94% | 8/3.81% | 7/13.73% | 13/12.04% | 9/6.98% | 7/5.04% | 8/4.28% | 10/5.35% | 24/10.96% | 33/10.09% | 27/7.71% | 70/8.18% | 222/8.23% |
|  | Nanotechnology | 3/1.47% | 3/1.43% | 1/1.96% | 2/1.85% | 9/6.98% | 3/2.16% | 5/2.67% | 16/8.56% | 24/11.42% | 25/7.65% | 44/12.57% | 94/10.98% | 230/8.53% |
|  | Physics | 8/3.92% | 9/4.29% | 6/11.76% | 6/5.56% | 13/10.08% | 14/10.07% | 23/12.30% | 13/6.95% | 25/7.31% | 33/10.09% | 17/4.86% | 13/1.52% | 171/6.34% |
|  | Statistics | 1/0.49% | 3/1.43% | 1/1.96% | 2/1.85% | 0/0.00% | 0/0.00% | 5/2.67% | 3/1.60% | 16/1.83% | 3/0.92% | 10/2.86% | 26/3.04% | 58/2.15% |
| Humanities (HUM) | total | 1/0.21% | 0/0.00% | 0/0.00% | 0/0.00% | 1/0.09% | 1/0.07% | 2/0.23% | 0/0.00% | 1/0.03% | 19/0.32% | 103/1.21% | 394/2.32% | 522/1.25% |
|  | Architecture | 1/100.00% | 0/0.00% | 0/0.00% | 0/0.00% | 0/0.00% | 0/0.00% | 0/0.00% | 0/0.00% | 0/0.00% | 3/15.79% | 6/5.83% | 24/6.09% | 35/6.70% |
|  | Arts - Film Studies | 0/0.00% | 0/0.00% | 0/0.00% | 0/0.00% | 0/0.00% | 0/0.00% | 0/0.00% | 0/0.00% | 0/0.00% | 0/0.00% | 3/2.91% | 10/2.54% | 13/2.49% |
|  | Arts - General | 0/0.00% | 0/0.00% | 0/0.00% | 0/0.00% | 0/0.00% | 0/0.00% | 0/0.00% | 0/0.00% | 0/0.00% | 7/36.84% | 34/33.01% | 194/49.24% | 235/45.02% |
|  | Arts - Music | 0/0.00% | 0/0.00% | 0/0.00% | 0/0.00% | 0/0.00% | 0/0.00% | 0/0.00% | 0/0.00% | 0/0.00% | 5/10.53% | 42/40.78% | 112/28.43% | 156/29.89% |
|  | Arts -Literature/  Poetry | 0/0.00% | 0/0.00% | 0/0.00% | 0/0.00% | 0/0.00% | 0/0.00% | 0/0.00% | 0/0.00% | 0/0.00% | 1/5.26% | 10/9.71% | 25/6.35% | 36/6.90% |
|  | Cartography | 0/0.00% | 0/0.00% | 0/0.00% | 0/0.00% | 1/100.00% | 0/0.00% | 0/0.00% | 0/0.00% | 0/0.00% | 0/0.00% | 1/0.97% | 0/0.00% | 2/0.38% |
|  | History - Africa | 0/0.00% | 0/0.00% | 0/0.00% | 0/0.00% | 0/0.00% | 0/0.00% | 0/0.00% | 0/0.00% | 0/0.00% | 0/0.00% | 1/0.97% | 0/0.00% | 1/0.19% |
|  | History - Asia | 0/0.00% | 0/0.00% | 0/0.00% | 0/0.00% | 0/0.00% | 0/0.00% | 0/0.00% | 0/0.00% | 1/100.00% | 5/26.32% | 2/1.94% | 10/2.54% | 19/3.64% |
|  | History - Europe | 0/0.00% | 0/0.00% | 0/0.00% | 0/0.00% | 0/0.00% | 0/0.00% | 0/0.00% | 0/0.00% | 0/0.00% | 0/0.00% | 0/0.00% | 1//0.25% | 2/0.38% |
|  | History - General | 0/0.00% | 0/0.00% | 0/0.00% | 0/0.00% | 0/0.00% | 0/0.00% | 0/0.00% | 0/0.00% | 0/0.00% | 0/0.00% | 1/0.97% | 1/0.25% | 2/0.38% |
|  | Journalism | 0/0.00% | 0/0.00% | 0/0.00% | 0/0.00% | 0/0.00% | 0/0.00% | 0/0.00% | 0/0.00% | 0/0.00% | 1/5.26% | 1/0.97% | 12/3.05% | 14/2.68% |
|  | Philosophy | 0/0.00% | 0/0.00% | 0/0.00% | 0/0.00% | 0/0.00% | 0/0.00% | 0/0.00% | 0/0.00% | 0/0.00% | 0/0.00% | 1/0.97% | 4/1.02% | 5/0.96% |
|  | Religion | 0/0.00% | 0/0.00% | 0/0.00% | 0/0.00% | 0/0.00% | 0/0.00% | 0/0.00% | 0/0.00% | 0/0.00% | 0/0.00% | 1/0.97% | 1/0.25% | 2/0.38% |
| Environmental Sciences (ENV) | total | 13/2.75% | 12/1.99% | 11/3.14% | 14/1.42% | 14/1.29% | 15/1.05% | 25/2.89% | 15/1.17% | 14/0.44% | 281/4.76% | 74/0.87% | 268/1.58% | 756/1.81% |
|  | Climate Change | 0/0.00% | 0/0.00% | 0/0.00% | 0/0.00% | 1/7.14% | 0/0.00% | 0/0.00% | 0/0.00% | 0/0.00% | 8/2.85% | 1/1.35% | 4/1.49% | 14/1.85% |
|  | Climatology | 0/0.00% | 1/8.33% | 4/9.09% | 0/0.00% | 0/0.00% | 0/0.00% | 4/16.00% | 1/6.67% | 2/14.29% | 48/17.08% | 3/4.05% | 0/0.00% | 60/7.94% |
|  | Ecology | 1/7.69% | 2/16.67% | 2/18.18% | 0/0.00% | 7/28.57% | 4/26.67% | 0/12.00% | 0/0.00% | 2/14.29% | 50/17.79% | 21/28.38% | 47/17.54% | 136/17.99% |
|  | Environmental  Sciences | 9/69.23% | 6/50.00% | 4/36.36% | 7/50.00% | 4/42.86% | 7/46.67% | 12/48.00% | 10/66.67% | 9/64.29% | 131/46.62% | 38/51.35% | 177/66.04% | 416/55.03% |
|  | Food Science | 2/15.38% | 1/8.33% | 1/9.09% | 1/7.14% | 4/7.14% | 2/13.33% | 1/4.00% | 1/6.67% | 0/00.00% | 5/1.78% | 4/5.41% | 16/5.97% | 35/4.63% |
|  | Ground/Surface  Water | 1/7.69% | 2/16.67% | 3/27.27% | 6/42.86% | 2/14.29% | 2/13.33% | 520.00% | 3/20.00% | 1/7.14% | 39/13.88% | 7/9.46% | 24/8.96% | 95/12.57% |
| Health Sciences (HSC) | total | 92/19.45% | 85/14.12% | 106/30.29% | 330/33.50% | 340/31.39% | 598/41.67% | 147/16.99% | 391/30.45% | 956/29.97% | 610/10.34% | 896/10.56% | 4388/25.80% | 8939/21.46% |
|  | Alternative | 4/4.35% | 1/1.18% | 2/1.89% | 0/0.00% | 16/4.71% | 7/1.17% | 4/2.72% | 6/1.53% | 9/0.94% | 8/1.31% | 18/2.01% | 124/2.83% | 199/2.23% |
|  | Anesthesia/  Anaesthesia | 2/2.17% | 1/1.18% | 1/0.94% | 3/0.91% | 4/1.18% | 2/0.33% | 2/1.36% | 1/0.26% | 9/0.94% | 8/1.31% | 14/1.56% | 71/1.62% | 118/1.32% |
|  | Biostatistics/  Epidemiology | 0/00.00% | 1/1.18% | 1/0.94% | 8/2.42% | 1/0.29% | 8/1.34% | 6/4.08% | 8/2.05% | 13/1.36% | 8/1.31% | 13/1.45% | 46/1.05% | 113/1.26% |
|  | Cardiology | 5/5.43% | 9/10.59% | 7/6.60% | 13/3.94% | 21/6.18% | 7/1.17% | 5/3.40% | 10/2.56% | 9/0.94% | 7/1.15% | 6/0.67% | 30/0.68% | 129/1.44% |
|  | Cardiovascular | 3/3.26% | 4/4.71% | 12/11.32% | 17/5.15% | 24/7.06% | 12/2.01% | 5/3.40% | 10/2.56% | 30/3.14% | 14/2.30% | 55/6.14% | 366/8.34% | 552/6.18% |
|  | Dentistry | 3/3.26% | 1/1.18% | 1/0.94% | 3/0.91% | 3/0.88% | 2/0.33% | 1/0.68% | 1/0.26% | 3/0.31% | 2/0.33% | 9/1.00% | 28/0.64% | 57/0.64% |
|  | Dermatology | 1/1.09% | 1/1.18% | 2/1.89% | 2/0.61% | 3/0.88% | 2/0.33% | 2/1.36% | 0/0.00% | 11/1.15% | 5/0.82% | 3/0.33% | 31/0.71% | 63/0.70% |
|  | Diabetes | 0/0.00% | 1/1.18% | 1/0.94% | 10/3.03% | 4/1.18% | 4/0.67% | 2/1.36% | 3/0.77% | 7/0.73% | 12/1.97% | 13/1.45% | 69/1.57% | 126/1.41% |
|  | Drug Design | 3/3.26% | 4/4.71% | 0/0.00% | 9/2.73% | 14/4.12% | 39/6.52% | 0/0.00% | 3/0.77% | 5/0.52% | 16/2.62% | 28/3.13% | 34/0.77% | 155/1.73% |
|  | Endocrinology | 2/2.17% | 3/3.53% | 3/2.83% | 19/5.76% | 7/2.06% | 5/0.84% | 0/0.00% | 3/0.77% | 17/1.78% | 12/1.97% | 8/0.89% | 67/1.53% | 146/1.63% |
|  | Gastroenterology | 3/3.26% | 3/3.53% | 5/4.72% | 11/3.33% | 13/3.82% | 55/9.20% | 9/6.12% | 67/17.14% | 91/9.52% | 52/8.52% | 29/3.24% | 156/3.56% | 494/5.53% |
|  | General | 0/00.00% | 2/2.35% | 0/00.00% | 3/0.91% | 2/0.59% | 1/0.17% | 0/0.00% | 1/0.26% | 6/0.63% | 9/1.48% | 31/3.46% | 119 /2.71% | 174/1.95% |
|  | Geriatric | 0/00.00% | 0/00.00% | 1/0.94% | 2/0.61% | 1/0.29% | 1/0.17% | 0/0.00% | 1/0.26% | 2/0.21% | 4/0.66% | 7/0.78% | 68/1.55% | 87/0.97% |
|  | Immunology | 6/6.52% | 7/8.24% | 6/5.66% | 9/2.73% | 7/2.06% | 12/2.01% | 5/3.40% | 7/1.79% | 9/0.94% | 4/0.66% | 19/2.12% | 106/2.42% | 197/2.20% |
|  | Infectious Disease | 5/5.43% | 1/1.18% | 3/2.83% | 7/2.12% | 4/1.18% | 6/1.00% | 3/2.04% | 9/2.30% | 28/2.93% | 21/3.44% | 33/3.68% | 59/1.34% | 197/2.00% |
|  | Internal | 0/0.00% | 0/0.00% | 0/0.00% | 6/1.82% | 0/0.00% | 2/0.33% | 0/0.00% | 0/0.00% | 4/0.42% | 4/0.66% | 6/0.67% | 65/1.48% | 87/0.97% |
|  | Neurology | 12/13.04% | 8/9.41% | 10/9.43% | 17/5.15% | 17/5.00% | 30/5.02% | 3/2.04% | 7/1.79% | 40/4.18% | 24/3.93% | 47/5.25% | 204/4.65% | 419/4.69% |
|  | Nursing | 0/0.00% | 0/0.00% | 0/0.00% | 0/0.00% | 1/0.29% | 1/0.17% | 0/0.00% | 1/0.26% | 2/0.21% | 0/00.00% | 23/2.57% | 190/4.33% | 218/2.44% |
|  | Nutrition | 0/0.00% | 0/0.00% | 0/0.00% | 2/0.61% | 1/0.29% | 0/0.00% | 1/0.68% | 0/00.00% | 0/00.00% | 3/0.49% | 6/0.67% | 25/0.57% | 38/0.43% |
|  | Obstetrics/  Gynecology | 2/2.17% | 0/0.00% | 6.60% | 11/3.33% | 7/2.06% | 17/2.84% | 5/3.40% | 16/4.09% | 70/7.32% | 33/5.41% | 35/3.91% | 207/4.72% | 410/4.59% |
|  | Occupational Health and Safety | 0/00.00% | 1/1.18% | 0/00.00% | 1/0.30% | 2/0.59% | 0/0.00% | 1/0.68% | 1/0.26% | 4/0.42% | 3/0.49% | 9/1.00% | 19/0.43% | 41/0.46% |
|  | Oncology | 8/8.70% | 5/5.88% | 14/13.21% | 54/16.36% | 60/17.65% | 143/23.91% | 10/6.80% | 75/19.18% | 312/32.64% | 140/22.95% | 108/12.05% | 466/10.62% | 1395/15.61% |
|  | Ophthalmology | 0/00.00% | 0/00.00% | 5/1.89% | 5/0.61% | 5/1.47% | 1/10.17% | 0/0.00% | 5/1.28% | 9/0.94% | 3/0.49% | 13/1.45% | 51/1.16% | 91/1.02% |
|  | Orthopedics | 2/2.17% | 0/00.00% | 2/1.89% | 21/6.36% | 6/1.76% | 20/3.34% | 10/6.80% | 17/4.35% | 50/5.23% | 35/5.74% | 35/3.91% | 183/4.17% | 381/4.26% |
|  | Otorhinolaryngology | 0/0.00% | 0/00.00% | 0/00.00% | 2/0.61% | 5/1.47% | 10/1.67% | 1/0.68% | 8/2.05% | 17/1.78% | 10/1.64% | 10/1.12% | 69/1.57% | 132/1.48% |
|  | Pathology | 0/0.00% | 2/2.35% | 2/1.89% | 14/4.24% | 27/7.94% | 101/16.89% | 3/2.04% | 0/00.00% | 3/0.31% | 0/0.00% | 3/0.33% | 1/0.02% | 156/1.75% |
|  | Pediatrics | 0/0.00% | 1/1.18% | 1/0.94% | 2/0.61% | 1/0.29% | 7/1.17% | /74.76% | 0/00.00% | 7/0.73% | 10/1.64% | 14/1.56% | 142/3.24% | 192/2.15% |
|  | Pharmacology | 3/3.26% | 9/10.59% | 8/7.55% | 12/3.64% | 28/8.24% | 19/3.18% | 9/6.12% | 8/2.05% | 19/1.99% | 23/3.77% | 49/5.47% | 236/5.38% | 423 /4.73% |
|  | Psychiatry | 0/00.00% | 0/00.00% | 0/00.00% | 1/0.30% | 2/0.59% | 3/0.50% | 2/1.36% | 2/0.51% | 6/0.63% | 9/1.48% | 5/0.56% | 2/10.48% | 51/0.57% |
|  | Public Health and Safety | 3/3.26% | 3/3.53% | 1/0.94% | 5/1.52% | 4/1.18% | 4/0.67% | 6/4.08% | 5/1.28% | 10/1.05% | 25/4.10% | 28/3.13% | 86/1.96% | 180/2.01% |
|  | Pulmonology | 5/5.43% | 0/0.00% | 2/1.89% | 11/3.33% | 10/2.94% | 9/1.51% | 4/2.72% | 20/5.12% | 43/4/.50% | 34/5.57% | 32/3.57% | 176/4.01% | 346/3.87% |
|  | Radiology/Imaging | 7/7.61% | 5/5.88% | 1/0.94% | 6/1.82% | 5/1.47% | 10/1.67% | 6/4.08% | 8/2.05% | 28/2.93% | 15/2.46% | 47/5.25% | 231/5.26% | 369/4.13% |
|  | Rehabilitation/  Therapy | 5/5.43% | 2/2.35% | 3/2.83% | 8/2.42% | 7/2.06% | 14/2.34% | 14/9.52% | 10/2.56% | 16/1.67% | 14/2.30% | 23/3.01% | 114/2.60% | 234/2.62% |
|  | Sports | 0/0.00% | 0/0.00% | 0/00.00% | 2/0.61% | 4/1.18% | 0/0.00% | 0/00.00% | 0/00.00% | 1/0.10% | 5/0.82% | 6/0.67% | 38/0.87% | 56/0.63% |
|  | Sports Science | 0/00.00% | 0/00.00% | 0/00.00% | 0/00.00% | 0/0.00% | 2/0.33% | 0/00.00% | 0/00.00% | 1/0.10% | 3/0.49% | 32/3.57% | 86/1.96% | 124/1.39% |
|  | Surgery | 3/3.26% | 5/5.88% | 8/7.55% | 19/5.76% | 16/4.71% | 18/3.01% | 12/8.16% | 40/10.23% | 19/1.99% | 14/2.30% | 48/5.36% | 278/6.34% | 480 /5.37% |
|  | Transplantation | 0/00.00% | 0/00.00% | 0/00.00% | 0/00.00% | 0/00.00% | 2/0.33% | 0/00.00% | 28/7.16% | 1/0.10% | 1/0.16% | 2/0.22% | 6/0.14% | 40/0.45% |
|  | Urology/Nephrology | 3/3.26% | 5/2.35% | 0/00.00% | 16/4.85% | 5/1.47% | 22/3.68% | 7/4.76% | 10/2.56% | 43/4.50% | 19/3.11% | 22/2.46% | 118/2.69% | 267/2.99% |
|  | Veterinary Science | 2/2.17% | 3/3.53% | 0/00.00% | 2/0.61% | 3/0.88% | 0/0.00% | 2/1.36% | 0/0.00% | 2/0.21% | 1/0.16% | 3/0.33% | 2/0.05% | 20/0.22% |
| Basic Life Sciences (BLS) | total | 109/23.04% | 119/19.77% | 157/44.86% | 484/49.14% | 467/43.12% | 651/45.37% | 428/49.48% | 545/42.45% | 1857/58.21% | 4045/68.57% | 3767/43.32% | 4400/25.87% | 16938/40.66% |
|  | Agriculture | 3/2.75% | 1/0.84% | 0/0.00% | 1/0.21% | 7/1.50% | 2/0.31% | 2/0.47% | 5/0.92% | 3/0.16% | 36/0.89% | 38/1.03% | 77/1.75% | 175/1.03% |
|  | Anatomy/Physiology | 2/1.83% | 1/0.84% | 4/2.55% | 10/2.07% | 8/1.71% | 7/1.08% | 12/2.80% | 7/1.28% | 12/0.65% | 11/0.27% | 16/0.44% | 6/0.14% | 96/0.57% |
|  | Anthropology | 0/0.00% | 0/0.00% | 0/0.00% | 0/0.00% | 0/0.00% | 0/0.00% | 0/0.00% | 0/0.00% | 0/0.00% | 0/0.00% | 0/0.00% | 1/0.02% | 1/0.01% |
|  | Archeology | 0/0.00% | 0/0.00% | 0/0.00% | 0/0.00% | 0/0.00% | 0/0.00% | 0/0.00% | 0/0.00% | 0/0.00% | 0/0.00% | 1/0.03% | 1/0.02% | 2/0.01% |
|  | Biochemistry | 17/15.60% | 14/11.76% | 21/13.38% | 30/6.20% | 47/10.06% | 51/7.83% | 92/21.50% | 135/24.77% | 378/20.36% | 431/10.66% | 262/7.13% | 528/12.00% | 2006/11.84% |
|  | Cancer | 4/3.67% | 13/10.92% | 23/14.65% | 67/13.84% | 70/14.99% | 165/25.35% | 49/11.45% | 81/14.86% | 375/20.19% | 899/22.22% | 832/22.63% | 798/18.14% | 3376/19.93% |
|  | Cellular | 21/19.27% | 21/17.65% | 35/22.29% | 101/20.87% | 99/21.20% | 91/13.98% | 116/27.10% | 164/30.09% | 531/28.59% | 1133/28.01% | 1154/31.39% | 1172/26.64% | 4638/27.38% |
|  | Forensic Sciences | 0/0.00% | 0/0.00% | 0/0.00% | 0/0.00% | 0/0.00% | 1/0.15% | 0/0.00% | 0/0.00% | 0/0.00% | 1/0.02% | 2/0.05% | 0/0.00% | 4/0.02% |
|  | General | 4/3.67% | 8/6.72% | 3/3.82% | 15/3.10% | 11/2.36% | 7/1.08% | 1/0.23% | 0/0.00% | 3/0.16% | 7/0.17% | 53/1.44% | 37/0.84% | 152/0.90% |
|  | Genetics | 15/13.76% | 16/13.45% | 16/10.19% | 100/20.66% | 86/18.42% | 146/22.43% | 53/12.38% | 70/12.84% | 369/19.87% | 901/22.27% | 851/23.15% | 1012/23.00% | 3635/21.46% |
|  | Microbiology | 5/4.59% | 4/3.36% | 10/6.37% | 18/3.72% | 17/3.64% | 8/1.23% | 14/3.27% | 15/2.75% | 27/1.45% | 40/0.99% | 36/0.98% | 53/1.20% | 247/1.46% |
|  | Molecular | 18/16.51% | 22/18.49% | 26/16.56% | 108/22.31% | 77/16.49% | 120/18.43% | 44/10.28% | 5/0.92% | 33/1.78% | 327/8.08% | 155/4.22% | 352/8.00% | 1287/7.60% |
|  | Neuroscience | 5/5.50% | 4/5.04% | 10/5.10% | 18/2.69% | 17/5.14% | 8/2.92% | 14/1.17% | 13/2.39% | 31/1.67% | 47/1.16% | 38/1.03% | 56/1.27% | 266/1.57% |
|  | Nutrition | 0/0.00% | 2/1.68% | 0/0.00% | 3/0.62% | 4/0.86% | 2/0.31% | 2/0.47% | 2/0.37% | 1/0.05% | 3/0.07% | 3/0.08% | 9/0.20% | 31/0.18% |
|  | Paleontology | 0/0.00% | 0/0.00% | 0/0.00% | 1/0.21% | 0/0.00% | 0/0.00% | 0/0.00% | 0/0.00% | 0/0.00% | 1/0.02% | 0/0.00% | 1/0.02% | 3/0.02% |
|  | Parasitology | 1/0.92% | 0/0.00% | 0/0.00% | 0/0.00% | 0/0.00% | 1/0.15% | 1/0.23% | 1/0.18% | 2/0.11% | 0/0.00% | 4/0.11% | 2/0.05% | 12/0.07% |
|  | Plant Biology/Botany | 9/8.26% | 4/0.84% | 4/2.55% | 4/0.83% | 12/2.57% | 10/1.54% | 16/3.74% | 17/3.12% | 14/0.75% | 35/0.87% | 36/0.98% | 78/1.77% | 236/1.39% |
|  | Toxicology | 2/1.83% | 4/3.36% | 4/0.64% | 10/2.07% | 0/0.00% | 15/2.30% | 21/4.91% | 25/4.59% | 74/3.98% | 168/4.15% | 171/4.65% | 199/4.52% | 690/4.07% |
|  | Zoology | 2/1.83% | 6/5.04% | 3/1.91% | 3/0.62% | 5/1.07% | 6/0.92% | 0/0.00% | 5/0.92% | 4/0.22% | 5/0.12% | 24/0.65% | 18/0.41% | 81/0.48% |
| Business and Technology (B/T) | total | 48/10.15% | 136/22.59% | 19/5.43% | 39/3.96% | 112/11.27% | 23/1.60% | 59/6.82% | 126/9.81% | 116/3.64% | 495/8.39% | 2651/31.24% | 4631/27.23% | 8465/20.32% |
|  | Business –  Accounting | 0/0.00% | 2/1.47% | 0/0.00% | 0/0.00% | 0/0.00% | 1/4.35% | 1/1.69% | 1/0.79% | 0/0.00% | 10/2.02% | 50/1.88% | 33/0.71% | 98/1.16% |
|  | Business –  Economics | 3/6.25% | 7/5.15% | 3/15.79% | 3/7.69% | 3/2.46% | 1/4.35% | 3/5.08% | 3/2.38% | 2/1.72% | 38/7.68% | 127/4.79% | 271/5.85% | 464/5.48% |
|  | Business –  General | 9/18.75% | 26/19.12% | 0/0.00% | 4/10.26% | 0/0.00% | 0/0.00% | 1/1.69% | 1/0.79% | 1/0.86% | 26/5.25% | 72/2.71% | 196/4.23% | 336/3.97% |
|  | Business –  Management | 8/16.67% | 45/33.09% | 1/5.26% | 2/5.13% | 0/0.00% | 0/0.00% | 0/0.00% | 2/1.59% | 1/0.86% | 15/3.03% | 107/4.03% | 177/3.82% | 358/4.23% |
|  | Business –  Manufacturing | 1/2.08% | 10/7.35% | 0/0.00% | 1/2.56% | 1/0.82% | 0/0.00% | 12/20.34% | 4/3.17% | 2/1.72% | 6/1.21% | 43/1.62% | 66/1.43% | 146/1.72% |
|  | Business –  Marketing | 0/0.00% | 1/0.74% | 0/0.00% | 1/2.56% | 0/0.00% | 0/0.00% | 0/0.00% | 0/0.00% | 1/0.86% | 5/1.01% | 32/1.21% | 51/1.10% | 91/1.08% |
|  | Business –  Public Relations | 0/0.00% | 0/0.00% | 0/0.00% | /0.00% | 0/0.00% | 0/0.00% | 0/0.00% | 0/0.00% | 0/0.00% | 0/0.00% | 3/0.11% | 4/0.09% | 7/0.08% |
|  | Computer Science | 9/18.75% | 8/5.88% | 10/52.63% | 14/35.90% | 53/43.44% | 11/47.83% | 18/30.51% | 54/42.86% | 59/50.86% | 150/30.30% | 862/32.48% | 828/17.88% | 2076/24.52% |
|  | Data Science | 0/0.00% | 0/0.00% | 0/0.00% | 0/0.00% | 0/0.00% | 0/0.00% | 0/0.00% | 0/0.00% | 0/0.00% | 0/0.00% | 148/5.58% | 1367/29.52% | 1515/17.90% |
|  | Foreign Aid | 0/0.00% | 0/0.00% | 0/0.00% | 0/0.00% | 0/0.00% | 1/4.35% | 0/0.00% | 0/0.00% | 2/1.72% | 0/0.00% | 0/0.00% | 1/0.02% | 4/0.05% |
|  | Government | 0/0.00% | 0/0.00% | 1/5.26% | 2/5.13% | 0/0.00% | 1/4.35% | 1/1.69% | 0/0.00% | 1/0.86% | 4/0.81% | 16/0.60% | 36/0.78% | 62/0.73% |
|  | International  Relations | 0/0.00% | 1/0.74% | 0/0.00% | 2/5.13% | 0/0.00% | 1/4.35% | 1/1.69% | 2/1.59% | 2/1.72% | 5/1.01% | 20/0.75% | 33/0.71% | 67/0.79% |
|  | Technology | 15/31.25% | /2820.59% | 4/21.05% | 10/25.64% | 63/51.64% | 5/21.74% | 17/28.81% | 51/40.48% | 36/31.03% | 187/37.78% | 1080/40.69% | 1375/29.69% | 2871/33.92% |
|  | Transportation | 0/0.00% | 4/2.94% | 0/0.00% | 0/0.00% | 1/0.82% | 1/4.35% | 2/3.39% | 4/3.17% | 4/3.45% | 13/2.63% | 36/1.36% | 86/1.86% | 151/1.78% |
|  | Urban Planning | 3/6.25% | 4/2.94% | 0/0.00% | 0/0.00% | 1/0.82% | 1/4.35% | 3/5.08% | 4/3.17% | 5/0.31% | 36/7.27% | 55/2.07% | 107/2.31% | 219/2.59% |
| Social Sciences (SOC) | total | 6/1.27% | 40/6.64% | 6/1.71% | 10/1.02% | 10/0.92% | 8/0.56% | 17/1.97% | 20/1.56% | 27/0.85% | 122/2.07% | 735/8.66% | 2069/12.17% | 3070/7.37% |
|  | Communications | 2/33.33% | 2/5.00% | 2/33.33% | 3/30.00% | 3/30.00% | 2/25.00% | 2/11.76% | 1/5.00% | 4/14.81% | 4/3.28% | 37/5.03% | 108/5.22% | 170/ 5.54% |
|  | Criminology | 0/0.00% | 0/0.00% | 0/0.00% | 0/0.00% | 0/0.00% | 0/0.00% | 0/0.00% | 0/0.00% | 0/0.00% | 0/0.00% | 2/0.27% | 7/0.34% | 9/0.29% |
|  | Education | 1/16.67% | 2/5.00% | 2/33.33% | 0/0.00% | 3/30.00% | 0/0.00% | 6/35.29% | 7/35.00% | 4/14.81% | 42/34.43% | 433/58.91% | 865/41.81% | 1365/44.46% |
|  | Ethics/Bioethics | 0/0.00% | 0/0.00% | 0/0.00% | 0/0.00% | 0/0.00% | 0/0.00% | 1/5.88% | 1/5.00% | 2/7.41% | 0/0.00% | 865/0.41% | 1/0.05% | 8/0.26% |
|  | Forensics | 0/0.00% | 0/0.00% | 0/0.00% | 0/0.00% | 0/0.00% | 0/0.00% | 0/0.00% | 1/5.00% | 2/7.41% | 1/0.82% | 1/0.14% | 1/0.05% | 6/0.20% |
|  | Law/Legal Issues | 0/0.00% | 0/0.00% | 0/0.00% | 0/0.00% | 0/0.00% | 0/0.00% | 0/0.00% | 1/5.00% | 0/0.00% | 2/1.64% | 14/1.90% | 37/1.79% | 54/1.76% |
|  | Linguistics | 0/0.00% | 1/2.50% | 0/0.00% | 0/0.00% | 0/0.00% | 0/0.00% | 0/0.00% | 0/0.00% | 0/0.00% | 6/4.92% | 35/4.76% | 196/9.47% | 238/7.75% |
|  | Military/Naval  Studies | 0/0.00% | 29/72.50% | 0/0.00% | 0/0.00% | 0/0.00% | 0/0.00% | 0/0.00% | 0/0.00% | 0/0.00% | 1/0.82% | 1/0.14% | 2/0.10% | 33/1.07% |
|  | Philosophy | 0/0.00% | 0/0.00% | 0/0.00% | 0/0.00% | 0/0.00% | 0/0.00% | 0/0.00% | 0/0.00% | 0/0.00% | 4/3.28% | 1/0.14% | 12/0.58% | 17/0.55% |
|  | Political Science | 0/0.00% | 1/2.50% | 0/0.00% | 2/20.00% | 0/0.00% | 0/0.00% | 1/5.88% | 0/0.00% | 0/0.00% | 0/0.00% | 22/2.99% | 123/5.94% | 149/4.85% |
|  | Psychology | 3/50.00% | 3/7.50% | 1/16.67% | 2/20.00% | 1/10.00% | 5/62.50% | 4/23.53% | 5/25.00% | 9/33.33% | 21/17.21% | 43/5.85% | 265/12.81% | 362/11.79% |
|  | Sexual And  Marital Studies | 0/0.00% | 0/0.00% | 0/0.00% | 0/0.00% | 0/0.00% | 0/0.00% | 1/5.88% | 0/0.00% | 4/3.70% | 2/1.64% | 1/0.14% | 0/0.00% | 5/0.16% |
|  | Sociology | 0/0.00% | 2/5.00% | 1/16.67% | 1/10.00% | 2/20.00% | 1/12.50% | 2/11.76% | 2/10.00% | 5/14.81% | 9/7.38% | 26/3.54% | 71/3.43% | 121/3.94% |
|  | Sports and  Recreation | 0/0.00% | 0/0.00% | 0/0.00% | 2/20.00% | 1/10.00% | 0/0.00% | 0/0.00% | 2/10.00% | 1/3.70% | 30/24.59% | 116/15.78% | 381/18.41% | 533/17.36% |

**Reference**

4.	Van Noorden R. More than 10,000 research papers were retracted in 2023 - a new record. Nature 2023;624(7992):479-481.

16.	Mallapaty S. China conducts first nationwide review of retractions and research misconduct. Nature 2024;626(8000):700-701.

36.	Cokol M, Iossifov I, Rodriguez Esteban R, Rzhetsky A. How many scientific papers should be re-tracted? EMBO reports 2007;8(5):422-423.

37.	Quan W, Chen B, Shu F. Publish or impoverish: An investigation of the monetary reward system of science in China (1999-2016). Aslib Journal of Information Management 2017;69(5):486-502.

38.	Guraya SY, Norman RI, Khoshhal KI, Guraya SS, Forgione A. Publish or Perish mantra in the medical field: A systematic review of the reasons, consequences and remedies. Pak J Med Sci 2016;32(6):1562-1567.

39.	Dohm L. Scientific Retractions May Become Easier to Spot As Retraction Watch Finds New Partner. In: Science; 2023.

40.	von Isenburg M, Oermann MH, Howard V. Plagiarism detection software and its appropriate use. Nurse Author & Editor 2019;29(1):1-10.

41.	Liverpool L. AI intensifies fight against 'paper mills' that churn out fake research. Nature 2023;618(7964):222-223.
