# Supplementary material for: Mapping retracted articles and exploring regional differences in China, 2012–2023
Source: PLoS One. 2024 Dec 2;19(12):e0314622. doi: 10.1371/journal.pone.0314622 (PMC11611127; doi:10.1371/journal.pone.0314622)
Supplement: Attachment — Submitted filename: Response to Reviewers.docx [file pone.0314622.s012.docx]

**Response to Reviewers**

**Dear Editor,**

We are grateful for the valuable comments and suggestions given by the reviewers concerning our manuscript entitled “**Mapping retracted articles and exploring regional differences in China, 2012-2023**” **(ID:** **PONE-D-24-27000R1).** We give our responses to the reviewer in this letter. We hope that after our replies, our manuscript will be considered for publication in **PLOS ONE**. The detailed responses are listed below, and revised parts of the manuscript are marked with red color.

**Question 1: Please review your reference list to ensure that it is complete and correct. If you have cited papers that have been retracted, please include the rationale for doing so in the manuscript text, or remove these references and replace them with relevant current references. Any changes to the reference list should be mentioned in the rebuttal letter that accompanies your revised manuscript. If you need to cite a retracted article, indicate the article’s retracted status in the References list and also include a citation and full reference for the retraction notice.**

**Response**: Thanks. We have carefully reviewed our reference list to ensure that all citations are complete and accurate. For any missing references, we have made the necessary additions, which are highlighted in red in the revised manuscript for your easy reference.

**Reviewer #1:**

**I have gone through the revised paper. You are to be congratulated for your research on such a pressing topic. In most retracted or retraction studies China happened to top the list of retraction. Hopefully, this paper will raise the authors', publishers', and policymakers' attention so that they can take the necessary action.**

**Response:** Thank you very much for your thoughtful review and constructive feedback. We sincerely appreciate your recognition of the importance of this research on such a pressing issue. Your insights and support have been invaluable in enhancing the quality of this work. Once again, thank you for your valuable input.

**Reviewer #2:**

**There are no comments to the author; the author made all the corrections as suggested. As stated in the review, the author implemented an analysis of prominent institutes, publishers, and authors, adhering to the suggestion to replace the term "retracted manuscripts" with "retracted articles." The author provided additional explanations for Figure 4 and incorporated the most recent references related to retracted articles. The author has implemented the suggestions and corrections mentioned in the review. The paper is recommended for publishing.**

**Response:** Thank you for your detailed and constructive feedback. We greatly appreciate your positive evaluation and support, as your feedback has been invaluable in helping us refine the paper. Once again, thank you for taking the time and effort to review our manuscript.

**Reviewer #3:**

**Question 1. In page 6, a sentence reads "The Retraction Watch Database was completely open and freely available on September 12, 2023." What is the relation of mentioning the date here? Because it is freely available. In my opinion, the entire paragraph is not required since it is repeated in the next paragraph.**

**Response:** Thanks for your suggestion. We have deleted the entire paragraph in our revised manuscript.

**Question 2. A sub-heading reads "Subject, institutes, and publishers Analysis of retracted articles in China" which should be revised.**

**Response:** Thanks for your suggestion. We have revised the subheading in the revised manuscripts, as follows: **Analysis of Subjects, Publishers, and Institutions Involved in Retracted Articles in China**

**Question 3. According to authors, the reasons have been categorized into nine and they have referred an previous article (refer page 11). Contrary to this, Table S5 caption reads "Standardized reason categorization corresponding to categorization employed by the Retraction Watch Database". Which is correct?**

**Response:** Thank you for your suggestion. In this study, we primarily standardized the retraction reasons obtained from the Retraction Watch Database ( for detailed reasons, please see: <https://retractionwatch.com/retraction-watch-database-user-guide/retraction-watch-database-user-guide-appendix-b-reasons/>) into nine distinct categories, as referred to the article https://doi.org/10.1007/s11192-021-03895-1. Since the categorization of retraction reasons in Retraction Watch Database has certain limitations, such as “investigations by company/institution”, “investigation by third party”, “investigation by journal/publisher”, “rogue editor”, which do not reflect actual retraction reasons, we have addressed these issues in our analysis. Table S5 presents the relationship between these standardized categories and the reasons provided by the Retraction Watch Database, for example, the first row outlines the classification of retraction reasons as defined in the Retraction Watch Database, which includes categories such as Plagiarism of Text, Plagiarism of Image, Plagiarism of Data, Plagiarism of Article, Euphemisms for Plagiarism, these categories are collectively categorized under the broader term 'Plagiarism' , as referenced in the article.
